# Respiratory support in paediatric patients with bronchiolitis: a systematic review and meta-analysis

**DOI:** 10.1183/16000617.0223-2025

**Published:** 2026-07-01

**Authors:** Spyridon Karageorgos, Anastasios Panagiotis Chantzaras, Aris P. Agouridis, Aidan Beegan, Nicola O'Shea, Tessa Davis, Michael Barrett

**Affiliations:** 1 Faculty of Medicine and Dentistry, Blizard Institute, Queen Mary University of London, London, UK; 2 Pediatric Emergency Department, Aghia Sophia Children's Hospital, National and Kapodistrian University of Athens, Athens, Greece; 3 Department of Pediatric Respiratory Medicine, Royal Brompton Hospital, London, UK; 4 School of Medicine, European University Cyprus, Nicosia, Cyprus; 5 Department of Internal Medicine, German Medical Institute, Limassol, Cyprus; 6 Research and Innovation, Children's Health Ireland, Dublin, Ireland; 7 Library and Information Service, Children's Health Ireland, Dublin, Ireland; 8 Paediatric Emergency Research and Innovation (PERI), Department of Paediatric Emergency Medicine, Children's Health Ireland, Dublin, Ireland; 9 Women's and Children's Health, School of Medicine, University College Dublin, Dublin, Ireland

## Abstract

**Background:**

Recent evidence suggests that high-flow nasal cannula (HFNC) may confer better clinical outcomes compared with other respiratory support modalities in paediatric patients with bronchiolitis.

**Methods:**

We performed a literature search up to 21 January 2024 on PubMed, Scopus, Cochrane Library, Embase, Cumulative Index to Nursing and Allied Health Literature, World Health Organization International Clinical Trials Registry Platform and Clinicaltrials.gov. Randomised controlled studies including paediatric patients aged ≤24 months with bronchiolitis comparing the use of HFNC *versus* low-flow nasal cannula (LFNC) or continuous positive airway pressure (CPAP) were included. Preferred Reporting Items for Systematic Reviews and Meta-Analyses guidelines were followed. Random-effect modelling was used to estimate pooled effects.

**Results:**

18 studies with 4094 patients were included. HFNC did not reduce the need for mechanical ventilation compared to LFNC (risk ratio 0.69, 95% CI 0.33–1.44; p=0.32) or CPAP (risk ratio 0.90, 95% CI 0.65–1.24; p=0.52). HFNC resulted in less treatment failure compared to LFNC (risk ratio 0.44, 95% CI 0.23–0.87; p=0.02), whereas there was no difference compared to CPAP (risk ratio 1.28, 95% CI 0.90–1.80; p=0.17). HFNC was associated with significant reduction in length of paediatric intensive care unit (PICU) stay compared to LFNC (mean difference (MD) −0.40 95% CI −0.78– −0.02; p=0.04), but not compared to CPAP (MD −0.24, 95% CI −0.91–0.43; p=0.47).

**Interpretation:**

HFNC significantly reduced treatment failure, length of PICU stay and total oxygen therapy duration compared to LFNC. No differences between HFNC and CPAP in primary and secondary outcomes. HFNC could be considered as initial treatment modality in paediatric patients with bronchiolitis, especially in patients requiring escalation of treatment from LFNC.

## Shareable abstract

**HFNC could be considered as an initial treatment modality in paediatric patients with bronchiolitis, especially in patients requiring escalation of treatment from LFNC** https://bit.ly/4tv20C1

## Introduction

Bronchiolitis is one of the most common respiratory tract infections and a common cause of hospital and paediatric intensive care unit (PICU) admission in infants [1]. There are different definitions used worldwide, as in the United States of America bronchiolitis is a term used in infants aged ≤24 months, whereas in the United Kingdom/Ireland and Europe, it is a term used in children aged ≤12 months [1–4]. A proportion of paediatric patients presenting with bronchiolitis, especially those with moderate and severe clinical manifestations, may need respiratory support and potential PICU admission [1, 2]. There are currently multiple different respiratory support modalities available worldwide including low-flow nasal cannula (LFNC) or standard oxygen therapy, high-flow nasal cannula (HFNC), continuous positive airway pressure (CPAP), biphasic positive airway pressure (BiPAP) and mechanical ventilation [5–7]. During the past decade multiple studies focused on the optimisation of respiratory support in infants with bronchiolitis with the target to prevent the need for intubation and PICU admission.

This study aims to systematically review and compare the clinical effectiveness and safety of different respiratory support modalities in paediatric patients aged ≤24 months with bronchiolitis.

## Methods

We performed a systematic review and meta-analysis. Study protocol was registered at PROSPERO (identifier number CRD42024504872). Our study followed the Preferred Reporting Items for Systematic Reviews and Meta-Analyses (PRISMA) guidelines for Meta-analysis (supplementary table e1) [8]. Participants/population, intervention, comparison and outcome components for this systematic review and meta-analysis are presented in table 1.

**TABLE 1:** Participants/population, intervention, comparison and outcome components for systematic review and meta-analysis

**Participants/population**	Infants aged 1–24 months who have received respiratory support for bronchiolitis
**Intervention**	HFNC
**Comparators**	LFNC, CPAP/NIV
**Primary outcomes**	Need for mechanical ventilation Treatment failure (as defined in each study)
**Secondary outcomes**	Admission to PICU Length of hospital stay Length of PICU stay Total oxygen therapy days Respiratory distress respiratory interventions and support Physiological and clinical (*e.g.* vital signs, general appearance) Feeding; nutrition and hydration status Resource use (hospital-related short-term) Adverse events Hospital mortality PICU mortality Pain and discomfort Quality of life
**Study design**	RCTs Quasi-RCTs

HFNC: high-flow nasal cannula; LFNC: low-flow nasal cannula; CPAP: continuous positive airway pressure; NIV: noninvasive ventilation; PICU: paediatric intensive care unit; RCT: randomised controlled trial.

### Search strategy

A literature search was organised by two authors with the assistance of a medical librarian between 1 December 2023 and 21 January 2024 on PubMed, Scopus, Cochrane Library, Embase and the Cumulative Index to Nursing and Allied Health Literature. The search also included the World Health Organization International Clinical Trials Registry Platform and Clinicaltrials.gov for potential included studies published from inception up to 21 January 2024. Articles published in English, French and Spanish were considered. References of included studies and prior reviews were also reviewed for potential missed studies. Search terms used in different databases, after communication with the medical librarian, are presented in supplementary eFile 1. If data were incomplete, personal communication with corresponding authors was undertaken.

### Inclusion criteria

Studies were included if they met the following criteria.

1) Randomised controlled trial (RCTs) and quasi-RCTs.
2) Included paediatric patients aged 1–24 months with bronchiolitis needing respiratory support (either LFNC, HFNC or CPAP). In case studies including paediatric patients aged >24 months, extraction of data was performed for paediatric patients age 1–24 months either directly or *via* communication with corresponding authors.
3) Assessed effectiveness of HFNC *versus* LFNC and/or CPAP.
4) Data regarding primary and/or secondary outcomes were available.

### Exclusion criteria

Studies meeting the following criteria were excluded from our analysis.

1) Non-RCTs, observational cohort studies, case series, case reports, conference abstracts.
2) Use of respiratory support for paediatric patients aged >24 months.
3) Did not meet the language inclusion criteria.
4) Data for paediatric patients aged ≤24 months could not be extracted (communication with study authors was performed before excluding the study).

### Outcome measures and data extraction

Two independent authors (S. Karageorgos and A.P. Chantzaras) reviewed and extracted relevant data from potentially included studies using Rayyan (https://www.rayyan.ai/). In case of potential disagreements, consensus with the participation of a third author (A.P. Agouridis) was reached. Data were extracted using an Excel (Microsoft Excel for Microsoft 365 MSO) spreadsheet organised *a priori*. Extracted data included the trial design, country, inclusion and exclusion criteria for the population of the study, patients’ characteristics (age, sex, prior medical history, preterm rate, duration of symptoms), setting of the trial (emergency department, PICU, paediatric ward). Data regarding study methodology were also extracted (randomisation and sampling methods). The interventions, primary and secondary outcomes of included studies were recorded. Finally, potential conflicts of interest were identified. Outcomes recorded were based on the recent Delphi survey and consensus workshop on developing a bronchiolitis core outcomes set [9].

### Risk of bias

We used the Cochrane Collaboration's risk of bias for randomised trials-2 (RoB-2) tool to assess risk of bias in included studies [10]. This tool assesses bias due to potential bias during the process of randomising patients in the trial; aberrations from the initial interventions; potential omitted or missing data on primary and secondary outcomes; bias in the way outcomes were measured in the trial; and selection bias of the reported result.

Each study was given an overall risk-of-bias score that was defined as low-risk, some concerns, or high-risk.

### Data synthesis and analysis

Categorical variables were presented as frequencies and percentages. Continuous variables were summarised as mean±sd. If medians and ranges or interquartile ranges were provided for continuous variables, we manually estimated the corresponding mean±sd using methods described in Hozo *et al*. [11] and Wan *et al*. [12]. Data tabulation and processing was in line with the principles described in the Cochrane Handbook [13]. We calculated the risk ratio based on the numbers of subjects and events in the arms of each study. To estimate the pooled risk ratios, mean differences and their 95% confidence intervals, we used random-effects meta-analysis using the DerSimonian and Laird [14] approach. Study proportion of variance in observed effects between studies and subgroups was assessed by using the I^2^ statistic (p-value cut-off=0.1). We used visual inspection of funnel plots to assess potential publication bias and small-study effects. We performed pairwise meta-analyses to compare treatments with available direct comparisons using random-effect models. Sensitivity analyses were decided *a priori* based on the potential study risk of bias. We performed a sensitivity analysis *via* excluding pilot studies and RCTs that were not completed, to minimise potential publication and reporting bias.

Meta-analysis was performed using Review Manager version 5.0. software. p<0.05 was considered significant.

## Results

### Literature search results

Literature review of databases identified a total of 9141 studies. After exclusion of duplicates, we double-screened 5636 studies, of which 119 were selected for full screening. Out of these, 18 studies were finally included in our study [15–32]. No publications were excluded from language restriction criteria. We contacted the authors of three studies [21, 28, 33] to enquire regarding data for paediatric patients aged ≤24 months. Data were provided for two of the studies [21, 28]. For one study, data on intention-to-treat and modified intention-to-treat for the primary and secondary outcomes were provided by the authors [28], and for one study authors provided raw data on paediatric patients [21]; hence, they were included in our meta-analysis. One study was excluded, since no data could be extracted and could not be provided in time by the authors [33]. Literature review process and reasons for exclusion are presented graphically in the PRISMA flowchart (figure 1). Risk-of-bias assessment using the RoB-2 tool was performed, and all studies were characterised as some concerns. Detailed data on risk of bias assessment are presented in supplementary figures e1 and e2. Potential publication bias was assessed using visual inspection of funnel plots for each outcome and are presented in supplementary figures e3–e12. Only one outcome (length of hospital stay between HFNC and LFNC) included 10 studies that are needed to assess for potential real asymmetry (supplementary figure e9).

**FIGURE 1:**
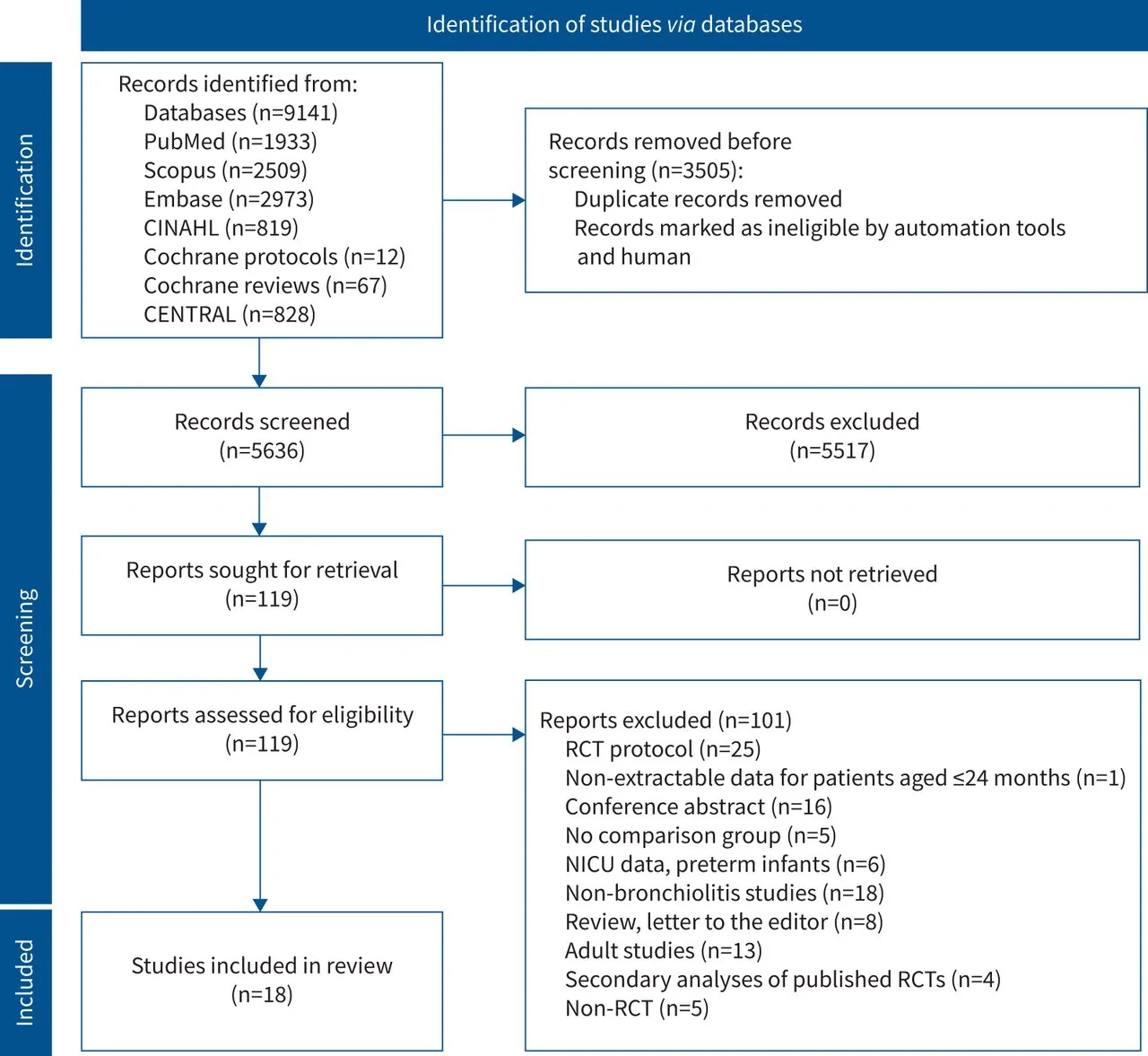
Preferred Reporting Items for Systematic Reviews and Meta-Analyses 2020 flow diagram. CINAHL: Cumulative Index to Nursing and Allied Health Literature; RCT: randomised controlled trial; NICU: neonatal intensive care unit.

### Characteristics of included studies

We included 18 RCTs in our meta-analysis with a total of 4094 paediatric patients aged ≤24 months with bronchiolitis. We identified 12 studies that compared HFNC to LFNC including data for 3273 patients (HFNC n=1633, LFNC n=1640) [17–25, 27, 30, 31]. Six studies with 821 patients compared the use of HFNC (n=415) *versus* CPAP (n=406) [15, 16, 26, 28, 29, 32].

11 studies were performed in emergency department/paediatric ward settings [17, 19–21, 23–25, 27, 30–32], whereas seven studies were performed in PICUs [15, 16, 18, 22, 26, 28, 29]. One study [19] was quasi-randomised and the remaining were RCTs. Three studies were pilot RCTs [23, 29, 34] and one study was terminated during enrolment [27]. One study was published in 2011 [23] and the rest between 2017 and 2023. All studies followed a parallel-group design. Funding source was reported in detail in 12 studies and potential conflicts of interest were included in all except one study [22]. Details of included studies characteristics are presented in table 2.

**TABLE 2:** Characteristics of included studies

First author, year [ref.]	Trial registration number	Sponsor	Conflict of interest reported	Location/country	Setting (ED/ward/PICU)	Study design	Study population, n
**Borgi, 2021** [15]	NCT04650230	NR	Yes	Tunis/Tunisia	PICU	RCT	255
**Cesar, 2020** [16]	NR	This study was supported by Hospital Infantil Sabará and Instituto PENSI. High-flow devices and circuits were provided by Vapotherm, Inc. at no cost to the investigators. Vapotherm was not involved in the planning, execution, data analysis, data interpretation, or writing of the manuscript, and was not privy to its results.	Yes	Sao Paolo/Brazil	PICU	Open-label, single-centre, RCT pilot trial	63
**Durand, 2019** [17]	NR	The study was funded by a grant from Programme Hospitalier de Recherche Clinique – PHRC 2016 (Ministère de la santé AORC 14056, P150931/IDRCB 2016-A00568–43). High-flow oxygen therapy devices and consumable materials were supplied by Fisher and Paykel Healthcare, Auckland, New Zealand. Funding information deposited with the Crossref Funder Registry.	Yes	Paris/France	ED and paediatric wards	Multicentre open-label RCT	268
**Ergul, 2018** [18]	NCT03342781	NR	Yes	Kayseri/Turkey	PICU	Single-centre, open, phase 4 RCT	60
**Eşki, 2022** [19]	NCT04245202	Supported by Ege University Research Foundation (grant/award number: ‘17-2.1/18').	Yes	Izmir/Turkey	Paediatric ward	Prospective, quasi-RCT	87
**Franklin, 2018** [20]	ACTRN12613000388718	Project grant (GNT1081736) from the NHMRC and by the Queensland Emergency Medical Research Fund. Regional site funding was obtained for Ipswich Hospital from the Ipswich Hospital Foundation and for the GCUH from the GCUH Foundation. The PREDICT sites were supported by a Centre for Research Excellence grant (GNT1058560) for paediatric emergency medicine from the NHMRC. Sites in Victoria, Australia, received infrastructure support from the Victorian Government Infrastructure Support Program, Melbourne. The Townsville Hospital was supported in part by a SERTA grant.	Yes	Australia and New Zealand	ED and paediatric wards	Multicentre RCT	1472
**Franklin, 2023** [21]	ACTRN12618000210279	This research was supported by grant 1139903 from the NHMRC in Australia and funding from the Thrasher Research Fund in USA, the Children's Hospital Foundation in Australia, the Perth Children's Hospital Foundation in Australia, and the Emergency Medicine Foundation in Australia. The OptiFlow equipment and consumables were supplied free of charge for this study by Fisher and Paykel Healthcare.	Yes	Australia and New Zealand	ED and paediatric ward	RCT	774
**Guna, 2023** [22]	NR	NR	NR	Madurai, Tamil Nādu, India	PICU	Prospective, parallel-group, open-label RCT	100
**Hilliard, 2012** [23]	NR	Two vapotherm devices were provided free of charge by the distributing company. There was no other funding for this study.	Yes	Bristol, UK	Paediatric respiratory medicine ward	Prospective, randomised, open pilot study	19
**Kepreotes, 2017** [24]	ACTRN12612000685819	Hunter Children's Research Foundation, John Hunter Hospital Charitable Trust, and the University of Newcastle Priority Research Centre GrowUpWell.	Yes	New South Wales, Australia	ED and paediatric wards	Open phase 4 RCT	202
**Kooiman, 2023** [25]	NCT02913040	Isala's Foundation for Innovation and Research funded this study with a grant of EUR 65 900 (grant number INNO 1621).	Yes	Netherlands	Paediatric ward	Multicentre, RCT	107
**Milési, 2017** [26]	NCT02457013	All phases were supported by Montpellier University Hospital (grant: research contract 2012–2015). The study was also supported by Fisher and Paykel Healthcare with the provision of 30 HFNC circuits. Fisher and Paykel was not involved in the study design and had no role in data management, data analysis and data interpretation, nor in the writing of the report and the decision to submit it for publication.	Yes	Montpellier, France	PICU	Prospective noninferiority RCT	142
**Murphy, 2020** [27]	NR	The consumables for the high-flow circuits were donated by Fisher and Paykel Healthcare, New Zealand.	Yes	Johannesburg, South Africa	High-care area/paediatric wards	Prospective RCT	28
**Ramnarayan, 2022** [28]	ISRCTN60048867	Funded by the NIHR Health Technology Assessment Programme (project number 17/94/28). Great Ormond Street Hospital for Children NHS Foundation Trust was the trial sponsor.	Yes	United Kingdom	PICU/HDU	Pragmatic, unblinded, multicentre, parallel-group, noninferiority trial	281
**Sarkar, 2018** [29]	NR	None	Yes	West Bengal/India	PICU	Single–centre, prospective, parallel group, open–label, and randomised pilot study	31
**Selvaraj, 2022** [30]	NR	None	Yes	Tamil Nadu/India	Paediatric ward	Open-label RCT	80
**Türe, 2020** [31]	NR	Costs were covered by the authors.	Yes	Konya/Turkey	ED	RCT	75
**Vahlkvist, 2019** [32]	NCT02618213	NR	Yes	Jutland, Denmark	Paediatric ward	RCT	50

ED: emergency department; PICU: paediatric intensive care unit; NR: not reported; RCT: randomised controlled trial; NHMRC: National Health and Medical Research Council; GCUH: Gold Coast University Hospital; PREDICT: Paediatric Research in Emergency Departments International Collaborative; SERTA: Study, Education, and Research Trust Account; NIHR: National Institute for Health and Care Research; HDU: high-dependency unit.

### Type of interventions

In the CPAP *versus* HFNC group, three studies compared CPAP to HFNC [16, 28, 32], two studies used nasal CPAP *versus* HFNC [26, 29] and one study used CPAP/noninvasive positive-pressure ventilation *versus* HFNC [15]. In the LFNC *versus* HFNC group, eight studies compared LFNC *versus* HFNC [17, 19–22, 24, 25, 27], two studies compared low-flow with the use of reservoir/non-rebreather mask *versus* HFNC [30, 31], one study compared low-flow provided *via* head box oxygen to HFNC [23] and one study compared low-flow *via* OxyMask to HFNC [18]. 11 studies provided data on their strategy regarding escalation of treatment [17, 20–22, 24, 26–29, 31, 32] and 11 studies reported their weaning strategy [15, 17–21, 23–25, 28, 31]. Strategies in both escalation and weaning protocols varied between studies.

### Primary outcomes

#### Need for mechanical ventilation

11 studies [17–23, 25, 27, 30, 31] with 3071 paediatric patients comparing the use of HFNC (n=1532) *versus* LFNC (n=1539) provided data on the need for mechanical ventilation. Use of HFNC did not reduce the need for mechanical ventilation (risk ratio 0.69, 95% CI 0.33–1.44; I^2^=42%, p=0.32; figure 2).

**FIGURE 2:**
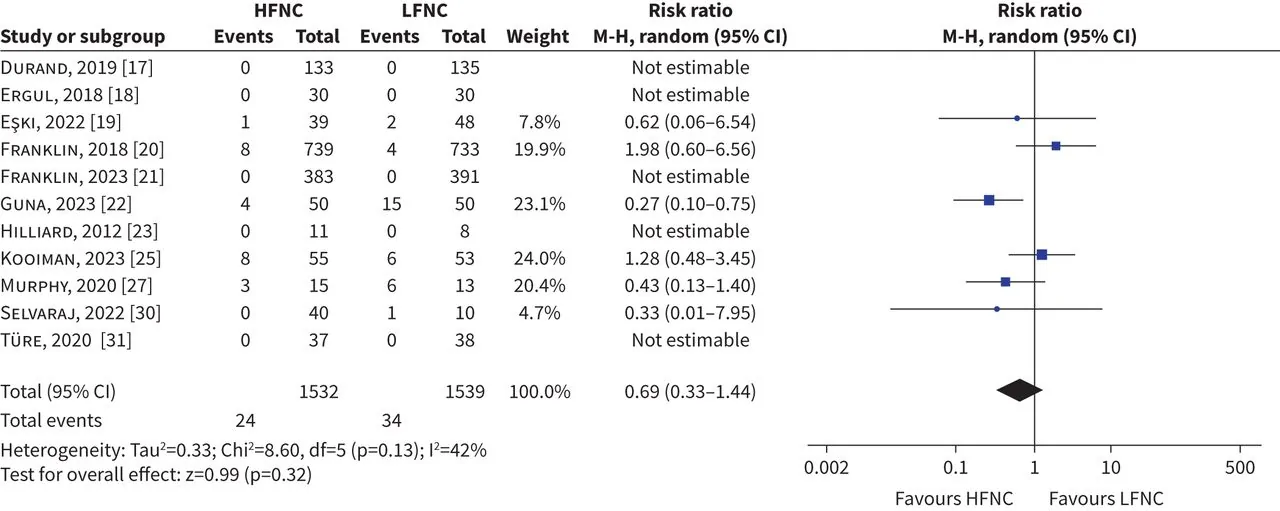
Forest plot of comparison: high-flow nasal cannula (HFNC) *versus* low-flow nasal cannula (LFNC); outcome: need for mechanical ventilation. M-H: Mantel–Haenszel; df: degrees of freedom.

Five studies [15, 16, 26, 28, 29] including 771 patients compared the use of HNFC (n=393) *versus* CPAP (n=378) regarding the need for mechanical ventilation. There was no difference between the two therapeutics approaches (risk ratio 0.90, 95% CI 0.65–1.24; I^2^=0%, p=0.52; figure 3).

**FIGURE 3:**
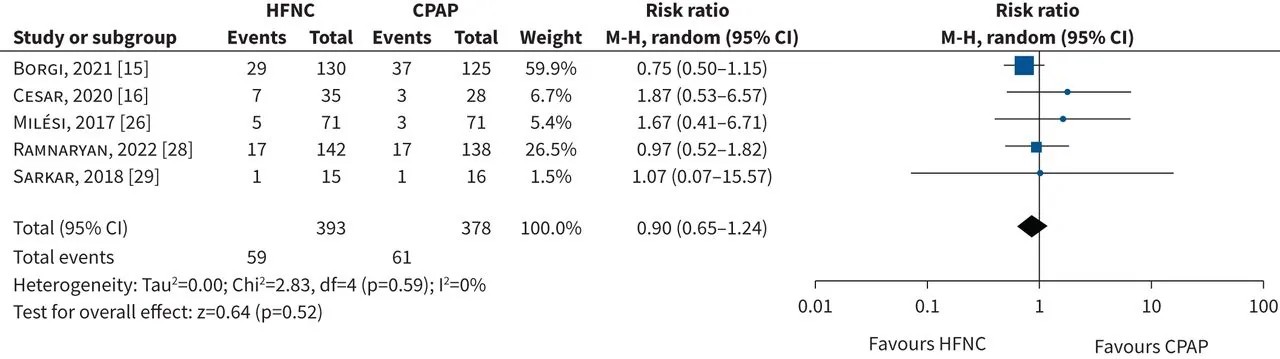
Forest plot of comparison: high-flow nasal cannula (HFNC) *versus* continuous positive airway pressure (CPAP); outcome: need for mechanical ventilation. M-H: Mantel–Haenszel; df: degrees of freedom.

#### Treatment failure

Treatment failure definition was available in 17 studies (supplementary table e2). 11 studies [17–24, 27, 30, 31] including 3165 patients (HFNC n=1578, LFNC n=1587) provided sufficient information regarding treatment failure. Use of HFNC was associated with significantly reduced treatment failure compared to the use of LFNC (risk ratio 0.44, 95% CI 0.23–0.87; I^2^=92%, p=0.02; figure 4a).

**FIGURE 4:**
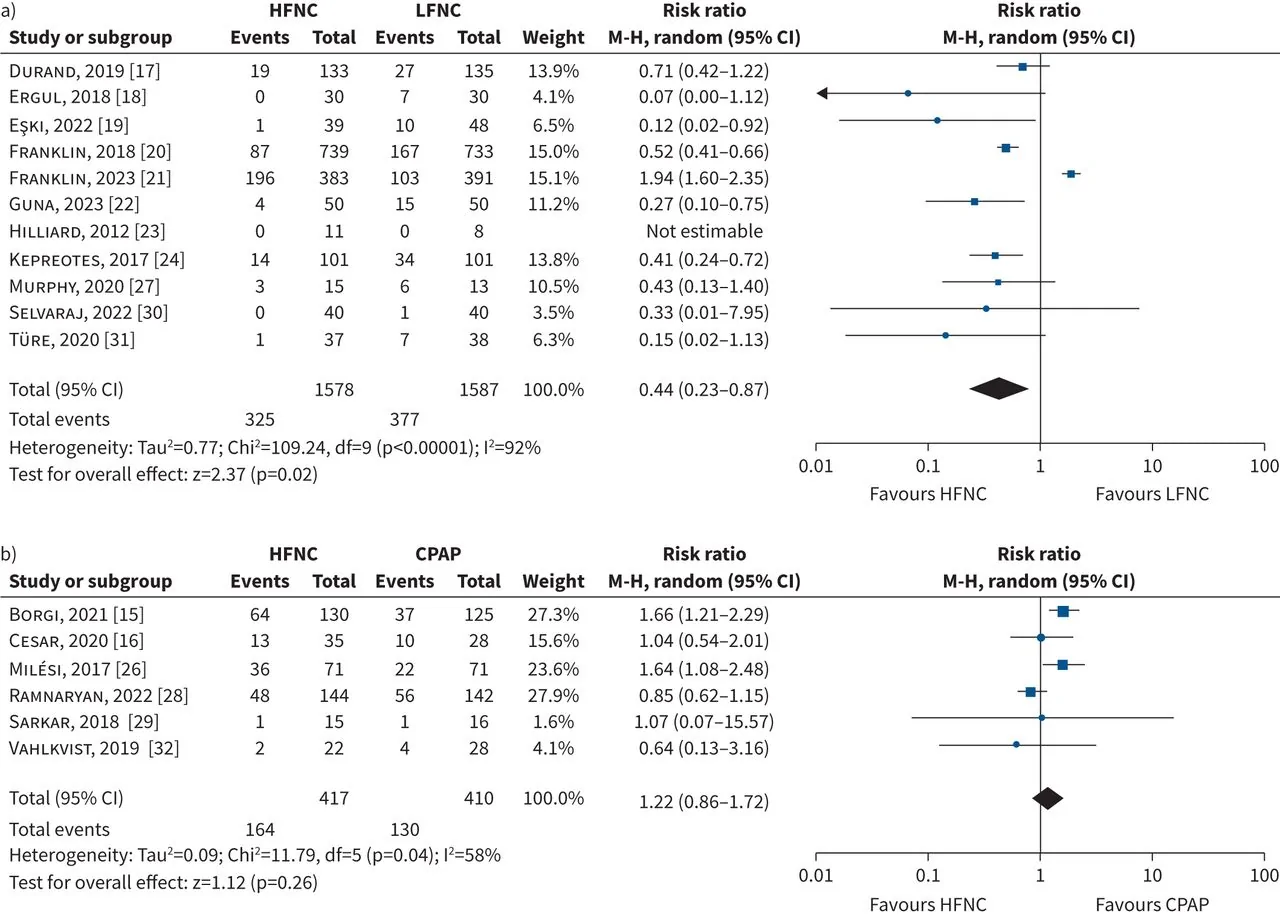
a) Forest plot of comparison: high-flow nasal cannula (HFNC) *versus* LFNC, outcome: treatment failure. b) Forest plot of comparison: HFNC *versus* continuous positive airway pressure (CPAP); outcome: treatment failure. M-H: Mantel–Haenszel; df: degrees of freedom.

Six studies [15, 16, 26, 28, 29, 32] comparing HFNC (n=417) *versus* CPAP (n=410) provided data on treatment failure. Use of HFNC was not associated with increased treatment failure (risk ratio 1.28, 95% CI 0.90–1.80; I^2^=57%, p=0.17; figure 4b).

### Secondary outcomes

#### Need for PICU admission

Eight studies [17, 19–21, 24, 25, 30, 31] with 3065 patients (HFNC n=1527, LFNC n=1538) provided data on admission of patients to PICU. There was no difference between the two treatment groups (risk ratio 1.22, 95% CI 0.93–1.60; I^2^=23%, p=0.16; supplementary figure e13).

#### Length of hospital stay

10 studies [17–21, 23–25, 30, 31] including 3144 patients compared data on length of hospital stay between HFNC (n=1568) *versus* LFNC (n=1576). Use of HFNC did not reduce the length of stay compared to LFNC (mean difference (MD) −0.31, 95% CI −0.72–0.09; I^2^=85%, p=0.13; supplementary figure e14).

Three studies [15, 16, 28] with 588 patients (HFNC n=300, CPAP n=288) reported data on the length of hospital stay. Use of HFNC was not associated with reduced length of hospital stay compared to use of CPAP (MD −0.38, 95% CI −1.75–0.98; I^2^=32%, p=0.58; supplementary figure e15).

#### Length of PICU stay

The use of HFNC was associated with a significant reduction in length of PICU stay in three studies including 2306 patients [18, 20, 21] (HFNC n=1152, LFNC n=1154) (MD −0.40, 95% CI −0.78– −0.02; I^2^=85%, p=0.04; supplementary figure e16).

Length of PICU stay data were reported in four studies comparing HFNC *versus* CPAP [16, 26, 28, 29], with 513 patients (HFNC n=262, CPAP n=251). There was no difference between treatments regarding the length of PICU stay (MD −0.24, 95% CI −0.91–0.43; I^2^=0%, p=0.47; supplementary figure e17).

#### Days of total oxygen therapy

Nine studies [17–20, 23–25, 30, 31] with data on 2370 patients compared the total oxygen therapy duration between HFNC (n=1185) and LFNC (n=1185). Use of HFNC was associated with a significant reduction in the duration of total oxygen therapy compared to LFNC (MD −0.37, 95% CI −0.63– −0.12; I^2^=81%, p=0.004; supplementary figure e18).

#### Adverse events

Data on adverse events were available in 11 out of 18 studies [15–17, 19–21, 24–26, 29, 31]. In three studies [21, 25, 31] there were no adverse events reported. In total, there were 28 adverse events reported in the HFNC group (apnoea n=8, abdominal distention n=3, pneumothorax n=4 (two of which were complicated by pneumomediastinum), epistaxis n=2, transient bradycardia n=3, desaturation due to disconnection n=1, inhalation injury n=1, skin lesions n=2, nasal injury n=4). In the CPAP group there were 34 adverse events reported (apnoea n=4, abdominal distention n=10, air leak (no additional information on the type of air leak were provided) n=2, skin lesions n=6, nasal injury n=12). Finally, there were seven adverse events in the LFNC group (apnoea n=3, pneumothorax n=1, transient bradycardia n=1, desaturation due to disconnection n=2).

#### Hospital and PICU mortality

16 studies provided data on hospital mortality [15–21, 23–27, 29–32]. There were no deaths reported in 1856 patients treated with HFNC, whereas two deaths were reported in 1589 patients treated with LFNC, and one death was reported in 268 patients in the CPAP group.

Regarding mortality in PICU, 10 studies provided available data [17–21, 23–26, 29]. There were no deaths in HFNC (n=1100), LFNC (n=953) and CPAP (n=117) groups.

#### Rescue therapy

11 studies included data on rescue therapy after initial treatment failure [15–17, 19–21, 24–26, 31, 32]. In seven studies with data on 344 HFNC patients who failed initial treatment [15–17, 21, 24, 26, 32], 125 were switched to LFNC, 68 switched to CPAP, 36 to nasal CPAP, 19 to noninvasive ventilation (NIV) and six to BiPAP. Additionally, 40 patients received other unspecified respiratory treatment and no data were reported for 50 patients. In seven studies with data on 350 LFNC patients [17, 19–21, 24, 25, 31], 207 were switched to HFNC, two to bubble CPAP, one to CPAP and one to NIV, whereas 20 received other respiratory support and there were no data for 119 patients. In three studies with data on 36 patients who required rescue therapy from CPAP [16, 26, 32], 24 received HFNC and seven switched to BiPAP. No data were provided for five patients.

#### Other outcomes

Six studies provided data regarding feeding method during respiratory support administration [15, 20, 21, 24, 25, 29]. Quality of life was assessed in two studies [19, 24], whereas it was mentioned as a secondary analysis plan in one study [28]. Pain and discomfort assessment was reported in five studies [25, 26, 28, 29, 32]. Only one study [24] included cost analysis data, which showed reduced cost regarding the consumables for LFNC compared to HFNC, but a higher cost in the LFNC when accounting for treatment failure and intensive care unit stay.

### Sensitivity analysis

After excluding three pilot studies [16, 23, 29] and one study that was terminated early due to results from another RCT that became available at the time of enrolment [27], results remained unchanged regarding primary and secondary outcomes (supplementary figures e19–e30).

## Discussion

Results of our meta-analysis showed that the use of HFNC was associated with a significantly reduced treatment failure, reduced length of PICU stay and reduced total oxygen therapy duration compared to the use of LFNC. However, there were no differences between the use of HFNC and CPAP in any of the primary and secondary outcomes in our analysis.

In our study, use of HFNC did not result in reduced need for mechanical ventilation compared to LFNC and CPAP. This is in line with results from the previous network meta-analysis that detected only a small difference in favour of HFNC in nonrandomised studies [5]. Of note, network meta-analysis results showed that NIV had the higher probability of being the most effective treatment in reducing mechanical ventilation (surface values under the cumulative ranking curve: NIV 57.03%, HFNC 50.87%, LFNC 42.11%) [5].

Regarding treatment failure, our results showed that HFNC reduced the risk of treatment failure compared to LFNC, whereas there was no difference between HFNC and CPAP groups. This corroborates data from previous studies that favoured HFNC *versus* LFNC, especially in studies including PICU patients [5]. In contrast, two previous meta-analyses on the basis of five studies comparing CPAP to HFNC showed a lower treatment failure rate in CPAP group [35, 36]. It should be noted that even though use of HFNC was associated with less treatment failure compared to LFNC, the clinical significance of this outcome is doubtful. This is because HFNC represents a step-up of treatment from LFNC, as also evident in our study where 59% patients who failed LFNC were rescued by HFNC. In addition, treatment failure was defined differently between included studies and was assessed objectively using different scales and clinical judgement by physicians who were not blinded to treatment intervention.

Comparing patients treated with HFNC with patients treated with LFNC, HFNC group had a shorter PICU stay duration based on three studies with available data. This may be potentially explained by the fact that in two studies 54% of patients initially treated with LFNC were switched to HFNC as an escalation therapy [20, 21]. Moreover, HFNC resulted in shorter duration of total oxygen therapy compared to LFNC, which is in concordance with results of the network meta-analysis [5].

It should be highlighted that there were no differences in either primary or secondary outcomes between HFNC and CPAP groups in our study. This is in contrast to previous meta-analyses that showed higher treatment failure in the HFNC group [36, 37]. This may be explained by the inclusion of a recent RCT [28] that increased the total number of patients included in the analysis. Also, only two studies had a significant difference favouring CPAP group [15, 26], whereas four studies did not show any difference [16, 28, 29, 32]. Lastly, the use of different devices and variable flow rates between studies should be considered while interpreting results of our study comparing HFNC to CPAP.

All treatments showed a favourable safety profile in our study. Adverse events were slightly more common in the CPAP group, especially regarding nasal injuries, compared to HFNC and LFNC. However, numbers were low and precluded us from performing a meta-analysis on this outcome. Importantly, both PICU and hospital mortality were rare events present in three out of 4094 included patients. This highlights the safety of available respiratory support modalities for paediatric patients aged ≤24 months presenting with bronchiolitis.

### Strengths

Our study has several strengths. Firstly, we performed an extensive literature search in multiple libraries and registries with the assistance of a medical librarian to reduce the possibility of missing a potentially included study. Secondly, we extracted data on primary and secondary outcomes based on the recommendations of the most recent bronchiolitis consensus workshop [9]. Communication with corresponding authors was performed when necessary to request data on paediatric patients aged ≤24 months. Our meta-analysis follows the PRISMA guidelines [38] and data handling was in line with the Cochrane Handbook. Finally, we performed a detailed assessment of the risk of bias of included studies [10].

### Limitations

There are multiple limitations to consider when interpreting results of our meta-analysis. Firstly, different methods of respiratory support were used between studies while escalation and weaning strategies also varied. Also, there was significant methodological heterogeneity between studies. Blinding was not possible due to the nature of intervention. Hence, detection and performance bias cannot be excluded. Moreover, generalisation of results may be limited, since 17 out of 18 included studies excluded patients with comorbidities (*e.g.* cardiac, pulmonary, neuromuscular disorders). Furthermore, treatment failure outcome was defined differently in studies. In addition, pilot studies may lead to imprecision, and RCTs that were terminated early may lead to reporting bias. However, our sensitivity analysis excluding these studies showed that results regarding both primary and secondary outcomes remained unchanged. Some studies did not include in their analysis outcomes regarding feeding, quality of care and cost analysis that are suggested by the recent bronchiolitis Delphi survey and consensus workshop as part of the core outcomes for bronchiolitis trials [9]. Future studies should focus on including these patient-centred outcomes in their protocol design and analysis. Finally, subgroup analysis based on the risk of bias could not be performed, as all studies were deemed to have some concerns in their study design.

### Conclusions

In this systematic review and meta-analysis, we showed that the use of HFNC was associated with a significantly reduced treatment failure, reduced length of PICU stay and reduced total duration of oxygen therapy compared to the use of LFNC. There were no differences between the use of HFNC and CPAP in the primary and secondary outcomes. In view of these results, HFNC could be considered as initial treatment modality in paediatric patients aged ≤24 months with bronchiolitis requiring respiratory support, especially in patients requiring escalation of treatment from LFNC.

**Points for clinical practice**

- Bronchiolitis is one of the most common respiratory tract infections and a common cause of hospital and PICU admission in infants.
- Initial optimal respiratory support is still under investigation.
- Results of this study showed that HFNC was associated with a significantly reduced treatment failure, reduced length of PICU stay and reduced total oxygen therapy duration compared to the use of LFNC.
- No differences between HFNC and CPAP.
- HFNC could be considered as initial treatment modality in paediatric patients aged ≤24 months with bronchiolitis requiring respiratory support, especially in patients requiring escalation of treatment from LFNC.

## Data Availability

This meta-analysis did not require the collection of new data but represents an analysis of previously published data. Data are available upon request to corresponding author.

## Supplementary material

**Please note:** supplementary material is not edited by the Editorial Office, and is uploaded as it has been supplied by the author. Supplementary material ERR-0223-2025.SUPPLEMENT
