# Supplementary material for: Respiratory support in paediatric patients with bronchiolitis: a systematic review and meta-analysis
Source: Eur Respir Rev. 2026 Jul 1;35(181):250223. doi: 10.1183/16000617.0223-2025 (PMC13320613; doi:10.1183/16000617.0223-2025)

Respiratory support in pediatric patients with Bronchiolitis: A Systematic Review and Meta-analysis – Supplemental Online Content

European Respiratory Review

This supplemental material is provided by the authors to provide readers additional information about their work.

Supplementary material contents

Supplementary eTable 1. PRISMA Checklist of Items to Include When Reporting A Systematic Review Involving Meta-analysis 4

Supplementary eTable 2. Treatment failure definition in included studies. 7

Supplementary eFile 1. Search Strategy for systematic review and network meta-analysis regarding the respiratory support for paediatric patients with bronchiolitis 11

Figure e1. Traffic light plot for risk of bias assessment of included studies using the Revised Cochrane risk-of-bias tool for randomized trials (RoB-2) 16

Figure e2. Summary plot for risk of bias assessment of included studies using the Revised Cochrane risk-of-bias tool for randomized trials (RoB-2) 17

Figure e3: Funnel plot of comparison: HFNC vs CPAP, outcome: Need for mechanical ventilation 18

Figure e4: Funnel plot of comparison: HFNC vs CPAP, outcome: Treatment failure 19

Figure e5: Funnel plot of comparison: HFNC vs LFNC, outcome: Need for mechanical ventilation 20

Figure e6: Funnel plot of comparison: HFNC vs LFNC, outcome: Treatment failure 21

Figure e7: Funnel plot of comparison: HFNC vs LFNC, outcome: Admission to PICU 22

Figure e8: Funnel plot of comparison: HFNC vs CPAP, outcome: Length of Hospital Stay (days) 23

Figure e9: Funnel plot of comparison: HFNC vs LFNC, outcome: Length of Hospital Stay (days) 24

Figure e10: Funnel plot of comparison: HFNC vs CPAP, outcome: Length of PICU Stay (days) 25

Figure e11: Funnel plot of comparison: HFNC vs LFNC, outcome: Length of PICU stay (days) 26

Figure e12: Funnel plot of comparison: HFNC vs LFNC, outcome: Total Oxygen Therapy (days) 27

Figure e13: Forest plot of comparison: HFNC vs LFNC, outcome: Admission to PICU 28

Figure e14: Forest plot of comparison: HFNC vs LFNC, outcome: Length of Hospital Stay (days)....29
Figure e15: Forest plot of comparison: HFNC vs CPAP, outcome: Length of Hospital Stay (days)....30
Figure e16: Forest plot of comparison: HFNC vs LFNC, outcome: Length of PICU stay (days). 31
Figure e17: Forest plot of comparison: HFNC vs CPAP, outcome: Length of PICU Stay (days). 32
Figure e18: Forest plot of comparison: HFNC vs LFNC, outcome: Total Oxygen Therapy (days)....33
Figure e19. **Forest plot of comparison: HFNC vs CPAP, outcome: Need for mechanical ventilation – sensitivity analysis** 34
Figure e20. **Funnel plot of comparison: HFNC vs CPAP, outcome: Need for mechanical ventilation – sensitivity analysis** 35
Figure e21**. Forest plot of comparison: HFNC vs CPAP, outcome: Treatment failure – sensitivity analysis** 36
Figure e22. **Funnel plot of comparison: HFNC vs CPAP, outcome: Treatment failure – sensitivity analysis**....37
Figure e23. **Forest plot of comparison: HFNC vs LFNC, outcome: Need for mechanical ventilation – sensitivity analysis** 38
Figure e24. **Funnel plot of comparison: HFNC vs LFNC, outcome: Need for mechanical ventilation – sensitivity analysis** 39
Figure e25. **Forest plot of comparison: HFNC vs LFNC, outcome: Treatment failure – sensitivity analysis** 40
Figure e26. **Funnel plot of comparison: HFNC vs LFNC, outcome: Treatment failure – sensitivity analysis** 41
Figure e27. **Forest plot of comparison: HFNC vs CPAP, outcome: Length of Hospital Stay (days) – sensitivity analysis** 42
Figure e28. **Funnel plot of comparison: HFNC vs CPAP, outcome: Length of Hospital Stay (days) – sensitivity analysis** 43

Figure e29. **Forest plot of comparison: HFNC vs CPAP, outcome: Length of PICU Stay (days) – sensitivity analysis** 44
Figure e30. **Funnel plot of comparison: HFNC vs CPAP, outcome: Length of PICU Stay (days) – sensitivity analysis** 45

Supplementary eTable 1. PRISMA Checklist of Items to Include When Reporting A Systematic Review Involving Meta-analysis

Section and Topic	Item #	Checklist item	Location where item is reported
**TITLE**			
Title	1	Identify the report as a systematic review.	1
**ABSTRACT**			
Abstract	2	See the PRISMA 2020 for Abstracts checklist.	6-7
**INTRODUCTION**			
Rationale	3	Describe the rationale for the review in the context of existing knowledge.	7-8
Objectives	4	Provide an explicit statement of the objective(s) or question(s) the review addresses.	8
**METHODS**			
Eligibility criteria	5	Specify the inclusion and exclusion criteria for the review and how studies were grouped for the syntheses.	10-11
Information sources	6	Specify all databases, registers, websites, organisations, reference lists and other sources searched or consulted to identify studies. Specify the date when each source was last searched or consulted.	9
Search strategy	7	Present the full search strategies for all databases, registers and websites, including any filters and limits used.	9 & supplementary 6-10
Selection process	8	Specify the methods used to decide whether a study met the inclusion criteria of the review, including how many reviewers screened each record and each report retrieved, whether they worked independently, and if applicable, details of automation tools used in the process.	10-11
Data collection	9	Specify the methods used to collect data from reports, including how many reviewers collected data from each report, whether they worked independently, any processes for obtaining or confirming data from	10-11

Section and Topic	Item #	Checklist item	Location where item is reported
process		study investigators, and if applicable, details of automation tools used in the process.	
Data items	10a	List and define all outcomes for which data were sought. Specify whether all results that were compatible with each outcome domain in each study were sought (e.g. for all measures, time points, analyses), and if not, the methods used to decide which results to collect.	9-11
	10b	List and define all other variables for which data were sought (e.g. participant and intervention characteristics, funding sources). Describe any assumptions made about any missing or unclear information.	11-12
Study risk of bias assessment	11	Specify the methods used to assess risk of bias in the included studies, including details of the tool(s) used, how many reviewers assessed each study and whether they worked independently, and if applicable, details of automation tools used in the process.	11
Effect measures	12	Specify for each outcome the effect measure(s) (e.g. risk ratio, mean difference) used in the synthesis or presentation of results.	11-12
Synthesis methods	13a	Describe the processes used to decide which studies were eligible for each synthesis (e.g. tabulating the study intervention characteristics and comparing against the planned groups for each synthesis (item #5)).	11-12
	13b	Describe any methods required to prepare the data for presentation or synthesis, such as handling of missing summary statistics, or data conversions.	11-12
	13c	Describe any methods used to tabulate or visually display results of individual studies and syntheses.	11-12
	13d	Describe any methods used to synthesize results and provide a rationale for the choice(s). If meta-analysis was performed, describe the model(s), method(s) to identify the presence and extent of statistical heterogeneity, and software package(s) used.	11-12
	13e	Describe any methods used to explore possible causes of heterogeneity among study results (e.g. subgroup analysis, meta-regression).	11-12
	13f	Describe any sensitivity analyses conducted to assess robustness of the synthesized results.	11-12
Reporting bias assessment	14	Describe any methods used to assess risk of bias due to missing results in a synthesis (arising from reporting biases).	11-12
Certainty assessment	15	Describe any methods used to assess certainty (or confidence) in the body of evidence for an outcome.	11-12
**RESULTS**			
Study selection	16a	Describe the results of the search and selection process, from the number of records identified in the search to the number of studies included in the review, ideally using a flow diagram.	12-14
	16b	Cite studies that might appear to meet the inclusion criteria, but which were excluded, and explain why	12-14

**Section and Topic**	**Item #**	**Checklist item**	**Location where item is reported**
		they were excluded.	
Study characteristics	17	Cite each included study and present its characteristics.	11-17
Risk of bias in studies	18	Present assessments of risk of bias for each included study.	Supplementary 11-12
Results of individual studies	19	For all outcomes, present, for each study: (a) summary statistics for each group (where appropriate) and (b) an effect estimate and its precision (e.g. confidence/credible interval), ideally using structured tables or plots.	15-20
Results of syntheses	20a	For each synthesis, briefly summarise the characteristics and risk of bias among contributing studies.	15-20
	20b	Present results of all statistical syntheses conducted. If meta-analysis was done, present for each the summary estimate and its precision (e.g. confidence/credible interval) and measures of statistical heterogeneity. If comparing groups, describe the direction of the effect.	15-20
	20c	Present results of all investigations of possible causes of heterogeneity among study results.	15-20
	20d	Present results of all sensitivity analyses conducted to assess the robustness of the synthesized results.	15-20 and supplementary 13-28
Reporting biases	21	Present assessments of risk of bias due to missing results (arising from reporting biases) for each synthesis assessed.	Not applicable
Certainty of evidence	22	Present assessments of certainty (or confidence) in the body of evidence for each outcome assessed.	15-20
**DISCUSSION**			
Discussion	23a	Provide a general interpretation of the results in the context of other evidence.	22-23
	23b	Discuss any limitations of the evidence included in the review.	23-24
	23c	Discuss any limitations of the review processes used.	23-24
	23d	Discuss implications of the results for practice, policy, and future research.	23-24
**OTHER INFORMATION**			
Registration and protocol	24a	Provide registration information for the review, including register name and registration number, or state that the review was not registered.	8
	24b	Indicate where the review protocol can be accessed, or state that a protocol was not prepared.	8
	24c	Describe and explain any amendments to information provided at registration or in the protocol.	8
Support	25	Describe sources of financial or non-financial support for the review, and the role of the funders or sponsors in the review.	Not applicable

Section and Topic	Item #	Checklist item	Location where item is reported
Competing interests	26	Declare any competing interests of review authors.	Not applicable
Availability of data, code and other materials	27	Report which of the following are publicly available and where they can be found: template data collection forms; data extracted from included studies; data used for all analyses; analytic code; any other materials used in the review.	Not applicable

Supplementary eTable 2. Treatment failure definition in included studies.

Study	Treatment failure definition
**Borgi et al (2021)**	FiO_2> 60% to maintain SpO_2≤ 90% or increasing of WOB.
**Cesar et al (2020)**	Determined by the care team and was defined as the need to escalate support to noninvasive bi-level pressure ventilation, or endotracheal intubation.
**Durand et al (2020)**	FiO_2 ≥ 40% or nasal flow oxygen > 2L min to maintain SpO_2 target, increasing m-WCAS score or > 5–6, refractory apnea and or increasing $PaCO_2$ at six hours.
**Ergul et al (2018)**	An escalation of care at any time point during the hospital stay. Fulfillment of 2 out of the three failure criteria<br>• No change or an increase in respiration rate compared to baseline.<br>• No change or an increase in heart rate compared to baseline.<br>• Persistence of low SpO_2 (< 92%) measurements despite an adequate oxygen flow rate and FiO_2 in the HFNC group/oxygen flow rate of 15 L/min in the mask group
**Eşki et al (2022)**	Treatment failure was considered at 4 hours when at least three of the following five criteria were present:<br>• An increase or no changes in HR relative to baseline<br>• An increase or no changes in RR relative to baseline<br>• Persistence of low SpO_2 (<92%) in the three near and independent measurements despite receiving

	FiO2 >40% (HFNCO) and at a maximum 10 L/min (LFO)<br>• An increase or no changes in CRS relative to baseline<br>• Arterial blood partial pressure of carbon dioxide >60 mm/Hg.
**Franklin et al (2018)**	Treatment failure that resulted in escalation of care during that hospital admission.<br><br>At the point of care, the treating clinicians determined the presence of treatment failure if at least three of four clinical criteria were met and clinicians decided that escalation of care was required. The criteria were as follows:<br><br>• heart rate remained unchanged or increased by any amount since admission (by contrast, a decrease of >5 beats per minute or into the normal range indicated treatment success)<br>• respiratory rate remained unchanged or increased by any amount since admission (by contrast, a decrease of >5 breaths per minute or into the normal range indicated treatment success)<br>• oxygen requirement in the high-flow group exceeded an Fio2 of at least 0.4 to maintain an oxygen-saturation level of at least 92% (or ≥94%, depending on the institution) or the requirement for supplemental oxygen in the standard-therapy group exceeded 2 liters per minute to maintain an oxygen-saturation level of at least 92% (or ≥94%)<br>• the hospital internal early-warning tool triggered a medical review and escalation of care.
**Franklin et al (2023)**	Escalation of care was recommended if the oxygen requirement in the high-flow therapy group exceeded an Fio_2 of 0.40 or 0.50 or the oxygen requirement in the standard oxygen group exceeded 2 L/min by nasal prong to maintain an Spo_2 of 90% or greater or exceeded 8 L/min by face mask to maintain an Spo_2 of 92% or greater.
**Guna et al (2023)**	If the children could not achieve any two or more of these criteria:<br><br>• RR decrease by 20 % or to normal.<br>• HR decrease by 20 % or to normal.<br>• FiO2 < 0.5, within first 2 hours of therapy were considered as treatment failure
**Hilliard et al (2011)**	Not requiring further respiratory support
**Kepreotes et al (2017)**	Critically abnormal observations that fell within the red zone on an age-appropriate SPOC for heart rate (age dependent), respiratory rate (age dependent), SpO2 (<90%), or respiratory distress score (severe) while on

	maximum therapy, along with a clinical decision by the treating physician or medical delegate that the current treatment was insufficient to reverse the deterioration. Red zone observations required a mandated rapid response within 10 min by the ICU team.
**Kooiman et al (2023)**	ND
**Milesi et al (2017)**	Any of the following:<br>• Rise in mWCAS<br>• Rise in RR<br>• Rise in EDIN score<br>• Apnea
**Murphy et al (2020)**	Therapy was terminated if any of the following conditions were met: impending respiratory failure (indicated by fatigue, apnoea, $PaCO_2$ >55 mmHg with pH <7.25), saturations <90% despite FiO_2 0.6, or altered mental state
**Ramnarayan et al (2022)**	FIO_2 ≥0.60, severe respiratory distress, patient discomfort, other
**Sarkar et al (2018)**	HR and/or RR remained unchanged/increased;<br>required FiO_2 >60% for nCPAP with PEEP >8; required<br>FiO_2 >60% for HHHFNC with maximum O_2 flow rate to<br>maintain SpO_2 >94% and no improvement or increase in<br>RDAI score.
**Selvaraj et al (2022)**	Escalation of respiratory support like invasive mechanical ventilation or admission into intensive care unit
**Türe et al (2020)**	Failure of vital signs to restore to age-adjusted normal range and of SpO_2 to return back to normal within 24 hours
**Vahlkvist et al (2019)**	Treatment failure was assessed by the physician in charge who also decided whether it should lead to a

	switch of system (CPAP to HFNC/HFNC to CPAP) or to transmission to the pediatric intensive care unit (PICU) with or without initiation of mechanical ventilation prior to transportation

Supplementary eFile 1. Search Strategy for systematic review and network meta-analysis regarding the respiratory support for paediatric patients with bronchiolitis

PubMed Search

("bronchioliti*"[All Fields] OR "wheez*"[All Fields] OR "metapneumovirus*"[All Fields] OR "para-influenza*"[All Fields] OR "parainfluenza*"[All Fields] OR "Respiratory syncytial"[All Fields] OR "RSV"[All Fields] OR "respiratory support"[All Fields] OR "bronchiolitis"[MeSH Terms] OR "metapneumovirus"[MeSH Terms] OR "parainfluenza virus 3, human"[MeSH Terms] OR "respiratory syncytial viruses"[MeSH Terms] OR "respiratory syncytial virus infections"[MeSH Terms] OR "respiratory insufficiency"[MeSH Terms]) AND ("high flow"[All Fields] OR "high-flow"[All Fields] OR "hfnc"[All Fields] OR "hhfnc"[All Fields] OR "hhhfnc"[All Fields] OR "hfnp"[All Fields] OR "hfot"[All Fields])

Search results: 1,933 (21st Jan)

Scopus Search

TITLE-ABS-KEY({bronchioliti*} OR {wheez*} OR {metapneumovirus*} OR {para-influenza*} OR {parainfluenza*} OR {Respiratory syncytial} OR {RSV} OR {respiratory support} OR {bronchiolitis} OR {metapneumovirus} OR {parainfluenza virus 3, human} OR {respiratory syncytial viruses} OR {respiratory syncytial virus infections} OR {respiratory insufficiency})

AND

TITLE-ABS-KEY({high flow} OR {high-flow} OR {hfnc} OR {hhfnc} OR {hhhfnc} OR {hfnp} OR {hfot})

Search results: 2,510 (21st Jan)

Database: CINAHL Complete via Ebscohost

Coverage: 1937-present

Search date: Monday, January 22, 2024 5:01:52 PM

#	Query	Limiters/Expanders	Last Run Via	Results

S1	bronchioliti* OR wheez* OR metapneumovirus* OR para influenza* OR parainfluenza* OR "Respiratory syncytial" OR "RSV" OR "respiratory support"	Search modes - Boolean/Phrase	Interface - EBSCOhost Research Databases<br>Search Screen - Advanced Search<br>Database - CINAHL Complete	13,414
S2	(MH "Bronchiolitis+") OR (MH "Respiratory Syncytial Viruses") OR (MH "Respiratory Syncytial Virus Infections") OR (MH "Respiratory Failure+")	Search modes - Boolean/Phrase	Interface - EBSCOhost Research Databases<br>Search Screen - Advanced Search<br>Database - CINAHL Complete	22,127
S3	S1 OR S2	Search modes - Boolean/Phrase	Interface - EBSCOhost Research Databases<br>Search Screen - Advanced Search<br>Database - CINAHL Complete	30,236
S4	"high flow" OR "high-flow" OR "hfnc" OR "hhfnc" OR "hhhfnc" OR "hfnp" OR "hfot"	Search modes - Boolean/Phrase	Interface - EBSCOhost Research Databases<br>Search Screen - Advanced Search<br>Database - CINAHL Complete	2,825
S5	S3 AND S4	Search modes - Boolean/Phrase	Interface - EBSCOhost Research Databases<br>Search Screen - Advanced Search<br>Database - CINAHL Complete	819

Database: Embase via Ovid

Coverage: 1974 to 22/01/2024

Search date: 22/01/2024

Query	Results
1. (bronchioliti* or wheez* or metapneumovirus* or para influenza* or parainfluenza* or "Respiratory syncytial" or "RSV" or "respiratory support").af.	118027
2. exp bronchiolitis/	26603
3. exp metapneumovirus/	4559
4. exp human parainfluenza virus 3/	956
5. exp respiratory syncytial virus infection/	8079
6. exp Human respiratory syncytial virus/	9027
7. exp respiratory failure/	131781
8. 1 or 2 or 3 or 4 or 5 or 6 or 7	243471
9. exp high flow nasal cannula therapy/	5978
10. ("high flow" or "high-flow" or "hfnc" or "hhfnc" or "hhhfnc" or "hfnp" or "hfot").af.	20557
11. 9 or 10	20626
12. 8 and 11	5195
13. limit 12 to "remove medline records"	2973

Database: Cochrane

Coverage: 1974 to 22/01/2024

Search date: 22/01/2024

Query	Results
#1<br><br>(bronchioliti* OR wheez* OR metapneumovirus* OR para influenza* OR parainfluenza* OR "Respiratory syncytial" OR "RSV" OR "respiratory support")	8618
#2<br><br>MeSH descriptor: [Bronchiolitis] explode all trees<br><br>MeSH	733
#3<br><br>MeSH descriptor: [Metapneumovirus] explode all trees<br><br>MeSH	8
#4<br><br>MeSH descriptor: [Parainfluenza Virus 3, Human] explode all trees<br><br>MeSH	18
#5<br><br>MeSH descriptor: [Respiratory Syncytial Viruses] explode all trees	301

MeSH	
#6<br><br>MeSH descriptor: [Respiratory Syncytial Virus Infections] explode all trees<br><br>MeSH	527
#7<br><br>MeSH descriptor: [Respiratory Insufficiency] explode all trees<br><br>MeSH	3959
#8<br><br>#1 or #2 or #3 or #4 or #5 or #6 or #7<br><br>Limits	12360
#9<br><br>"high flow" OR "high-flow" OR "hfnc" OR "hhfnc" OR "hhhfnc" OR "hfnp" OR "hfot"<br><br>Limits	3188
#10<br><br>#8 and #9<br><br>Limits	972

Search in World Health Organization International Clinical Trials Registry Platform and Clinicaltrials.gov will be based on the terms: “bronchiolitis” and “high-flow nasal cannula”.

Figure e1. Traffic light plot for risk of bias assessment of included studies using the Revised Cochrane risk-of-bias tool for randomized trials (RoB-2)

	D1	D2	D3	D4	D5	Overall
Borgi 2021	+	!	+	!	!	!
Cesar 2020	+	!	+	!	+	!
Durand 2020	+	!	!	!	+	!
Ergul 2019	+	!	!	!	+	!
Eşki 2022	!	!	+	!	!	!
Franklin 2018	+	!	+	!	+	!
Franklin 2023	+	+	+	!	+	!
Guna 2023	+	!	!	!	!	!
Hilliard 2011	!	!	+	!	!	!
Kepreotes 2017	+	+	+	!	!	!
Kooiman 2023	+	+	!	!	!	!
Milesi 2017	+	+	+	!	!	!
Murphy 2020	+	!	+	!	!	!
Ramnarayan 2022	+	+	+	!	+	!
Sarkar 2018	+	+	+	!	+	!
Selvaraj 2022	+	+	+	!	!	!
Türe 2020	+	!	+	!	!	!
Vahlkvist 2019	+	+	+	!	!	!

D1: Randomisation process
D2: Deviations from the intended interventions
D3: Missing outcome data
D4: Measurement of the outcome
D5: Selection of the reported result

Judgement

\+ Low risk

! Some concerns

\- High risk

Figure e2. Summary plot for risk of bias assessment of included studies using the Revised Cochrane risk-of-bias tool for randomized trials (RoB-2)

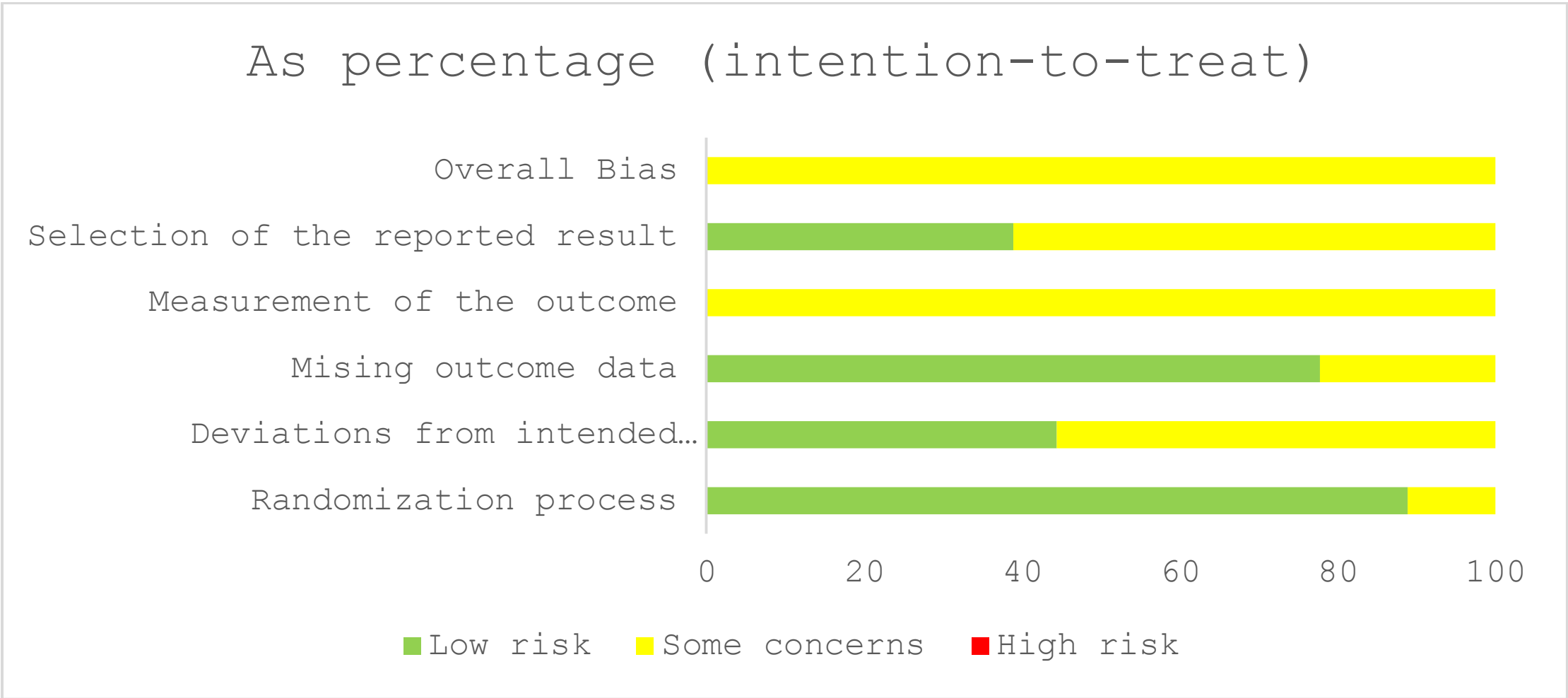

Figure e3: Funnel plot of comparison: HFNC vs CPAP, outcome: Need for mechanical ventilation

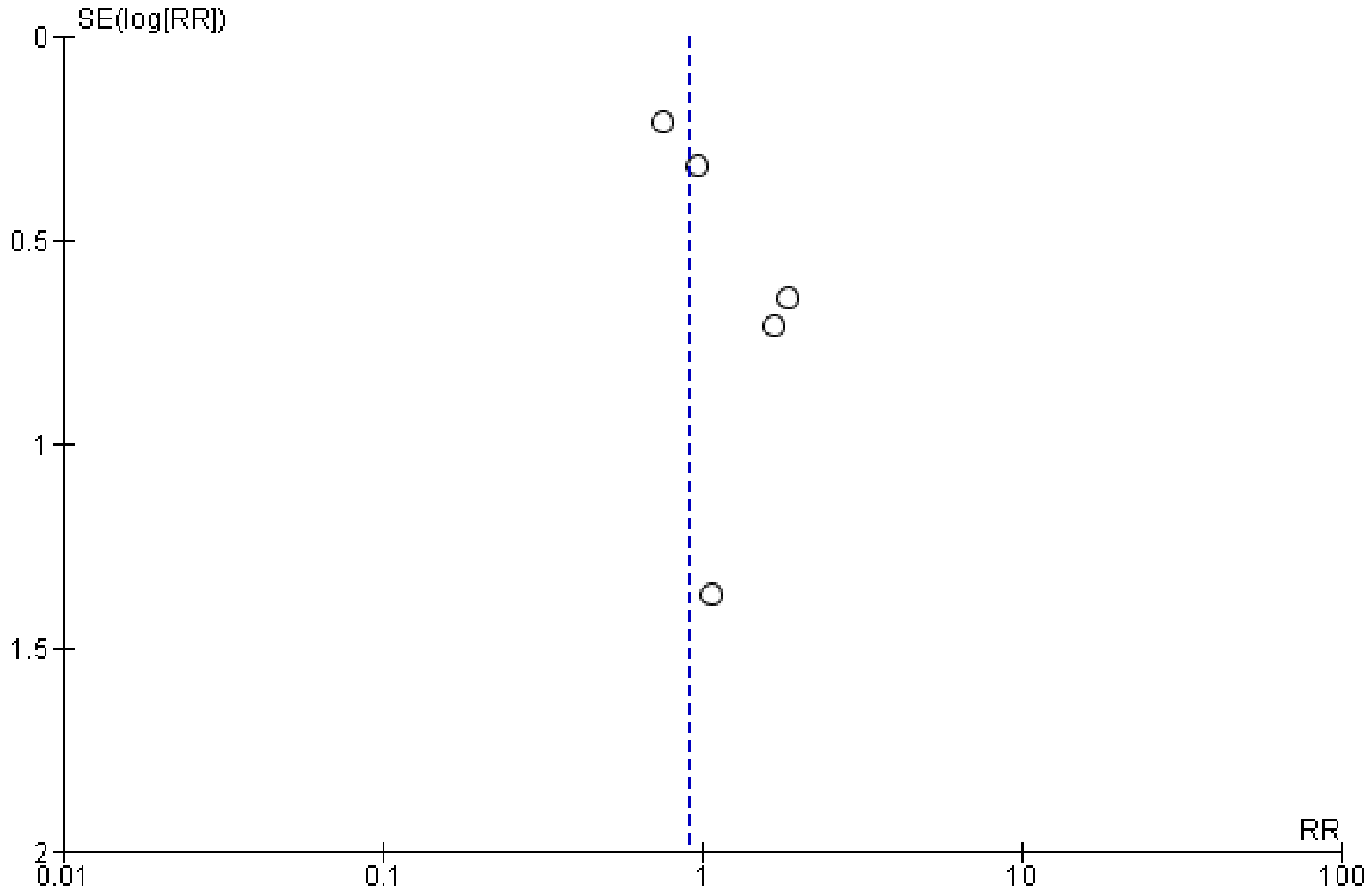

Figure e4: Funnel plot of comparison: HFNC vs CPAP, outcome: Treatment failure

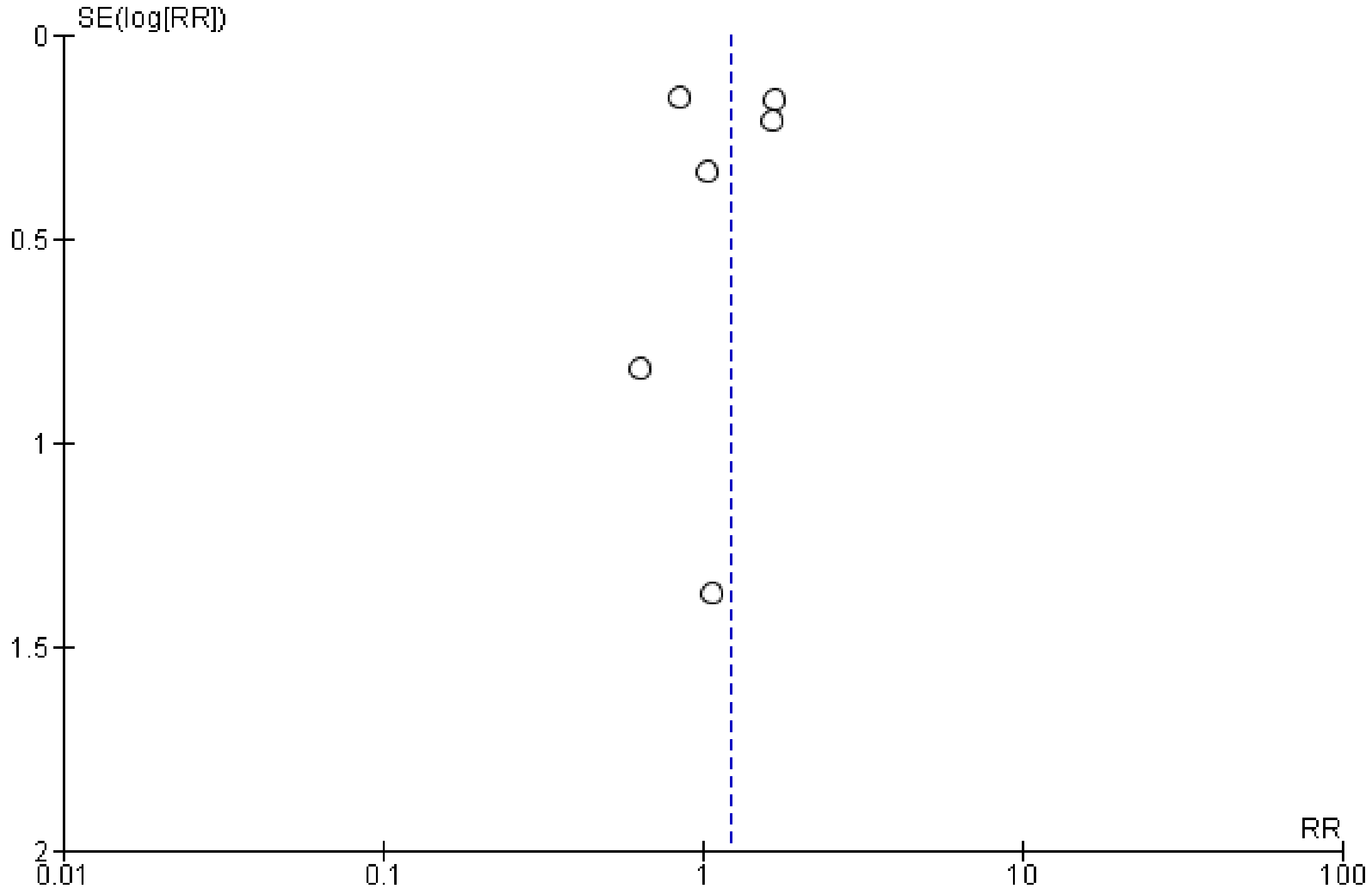

Figure e5: Funnel plot of comparison: HFNC vs LFNC, outcome: Need for mechanical ventilation

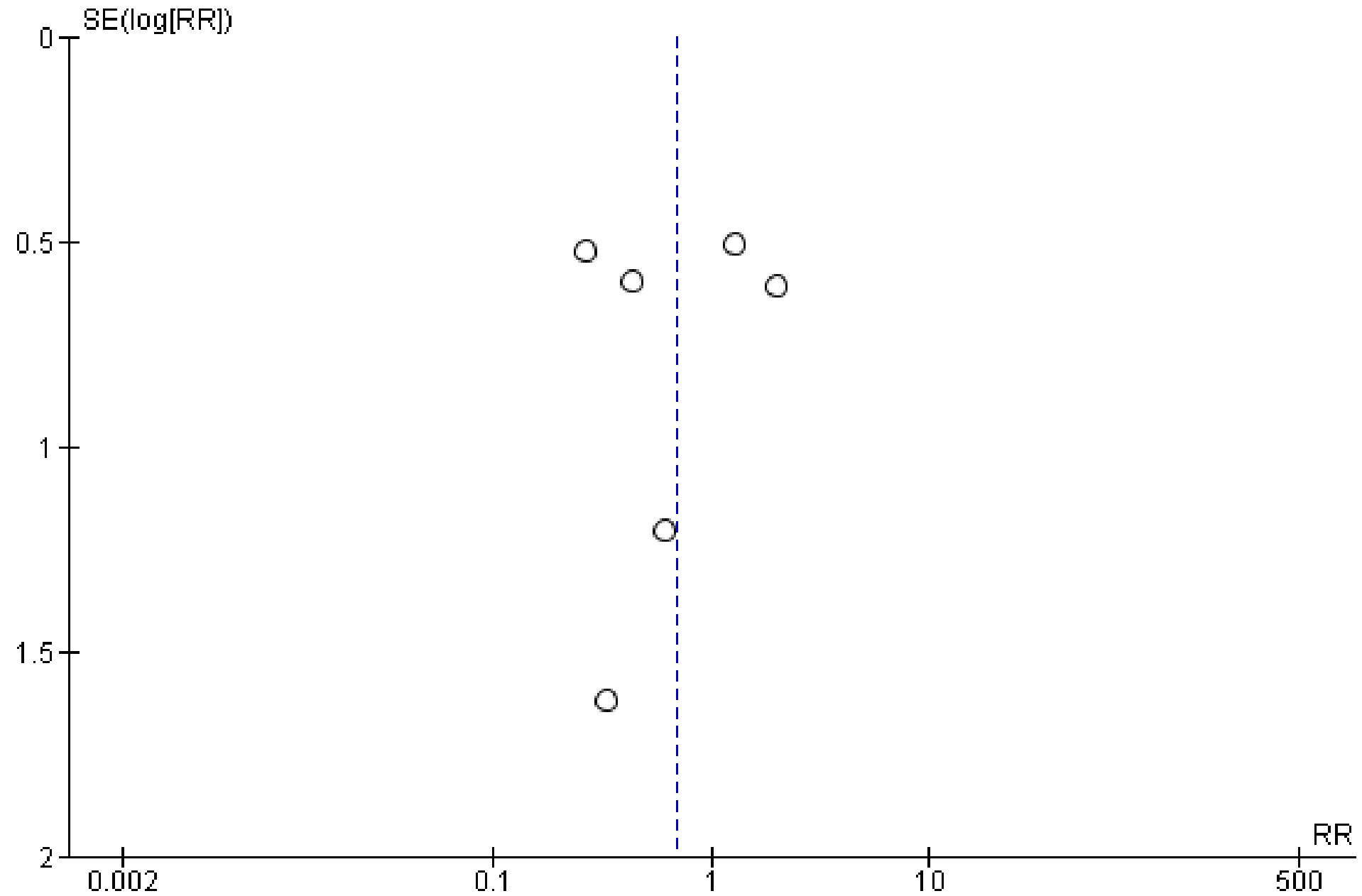

Figure e6: Funnel plot of comparison: HFNC vs LFNC, outcome: Treatment failure

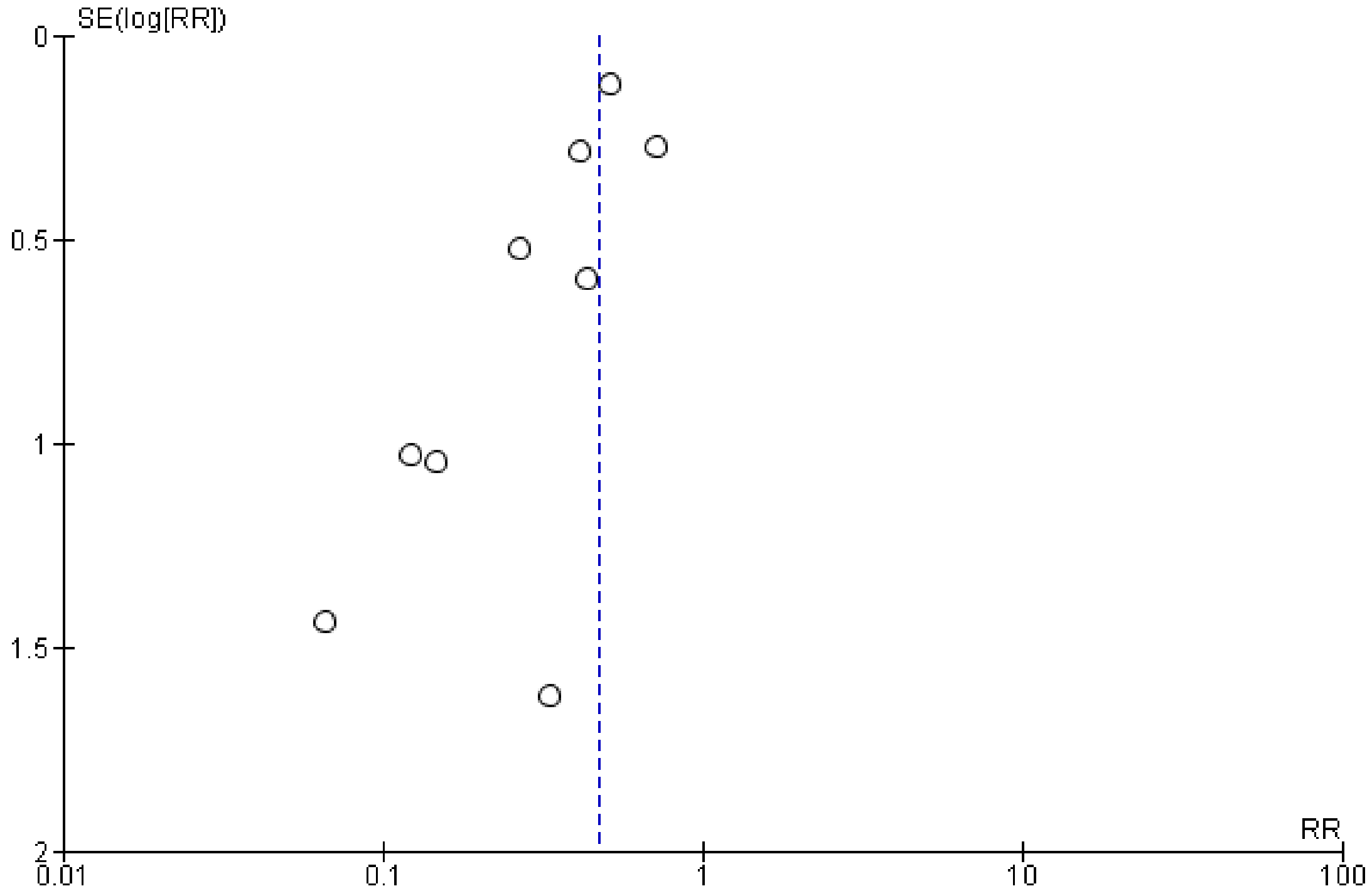

Figure e7: Funnel plot of comparison: HFNC vs LFNC, outcome: Admission to PICU

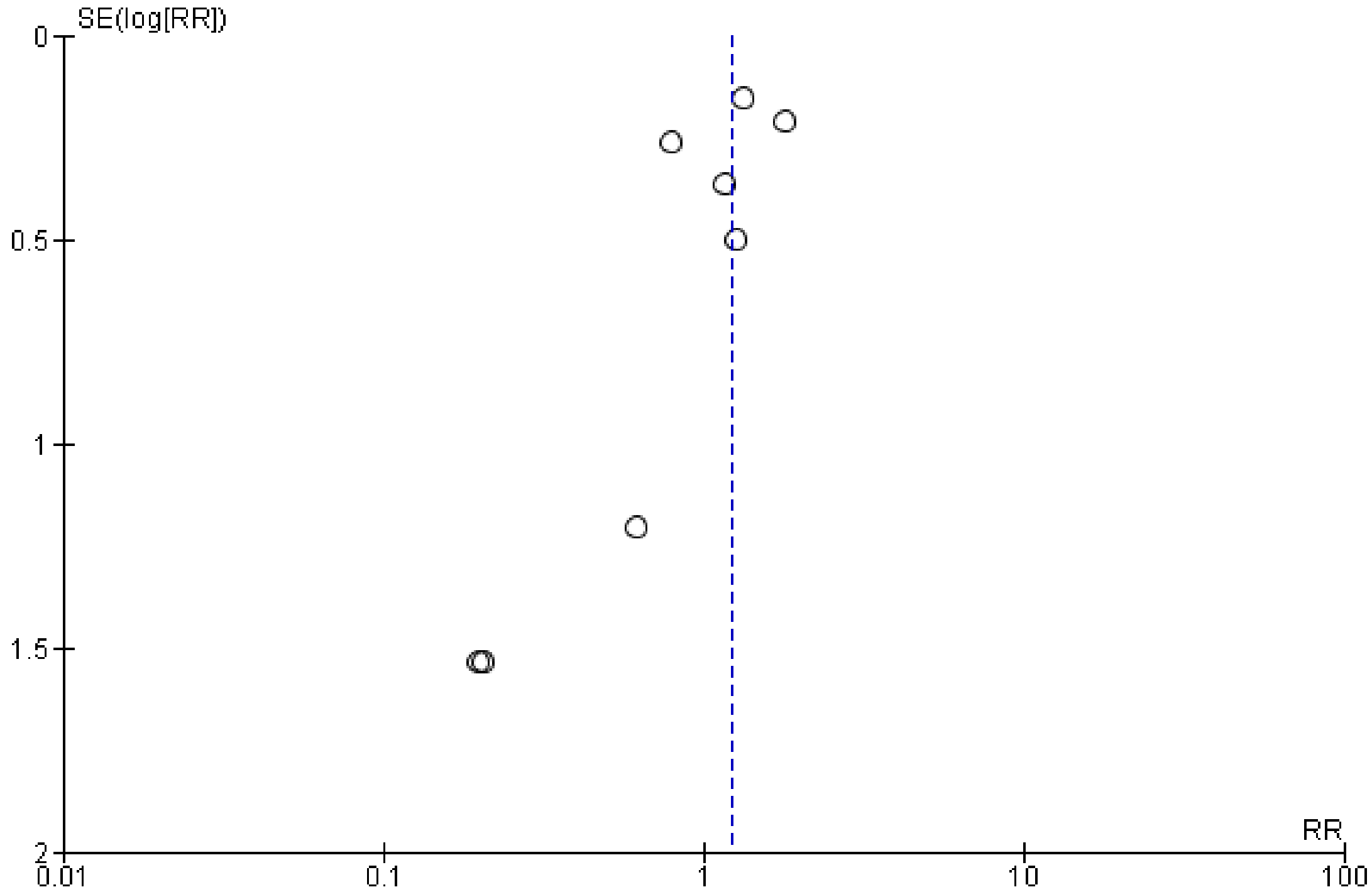

Figure e8: Funnel plot of comparison: HFNC vs CPAP, outcome: Length of Hospital Stay (days)

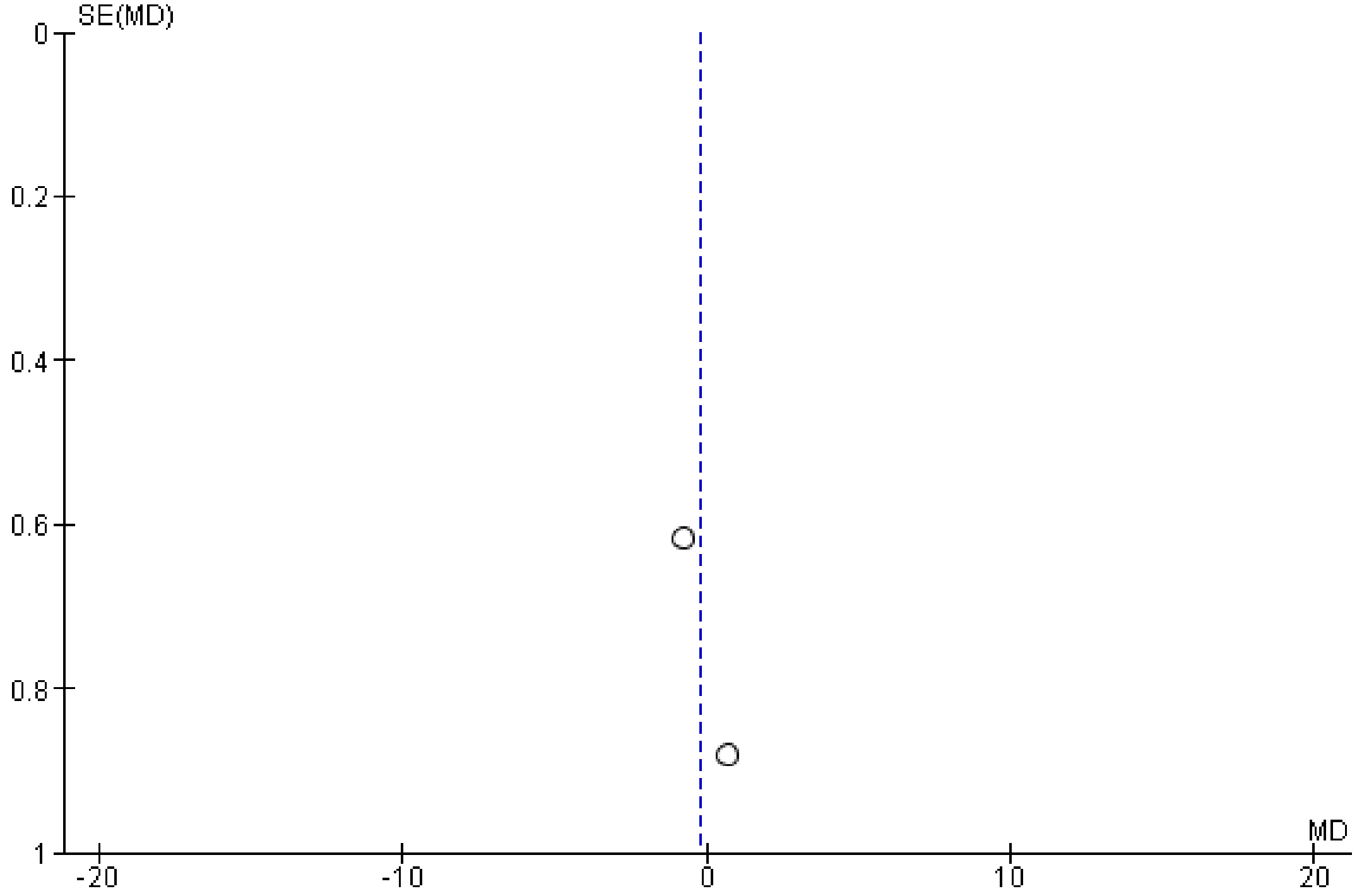

Figure e9: Funnel plot of comparison: HFNC vs LFNC, outcome: Length of Hospital Stay (days)

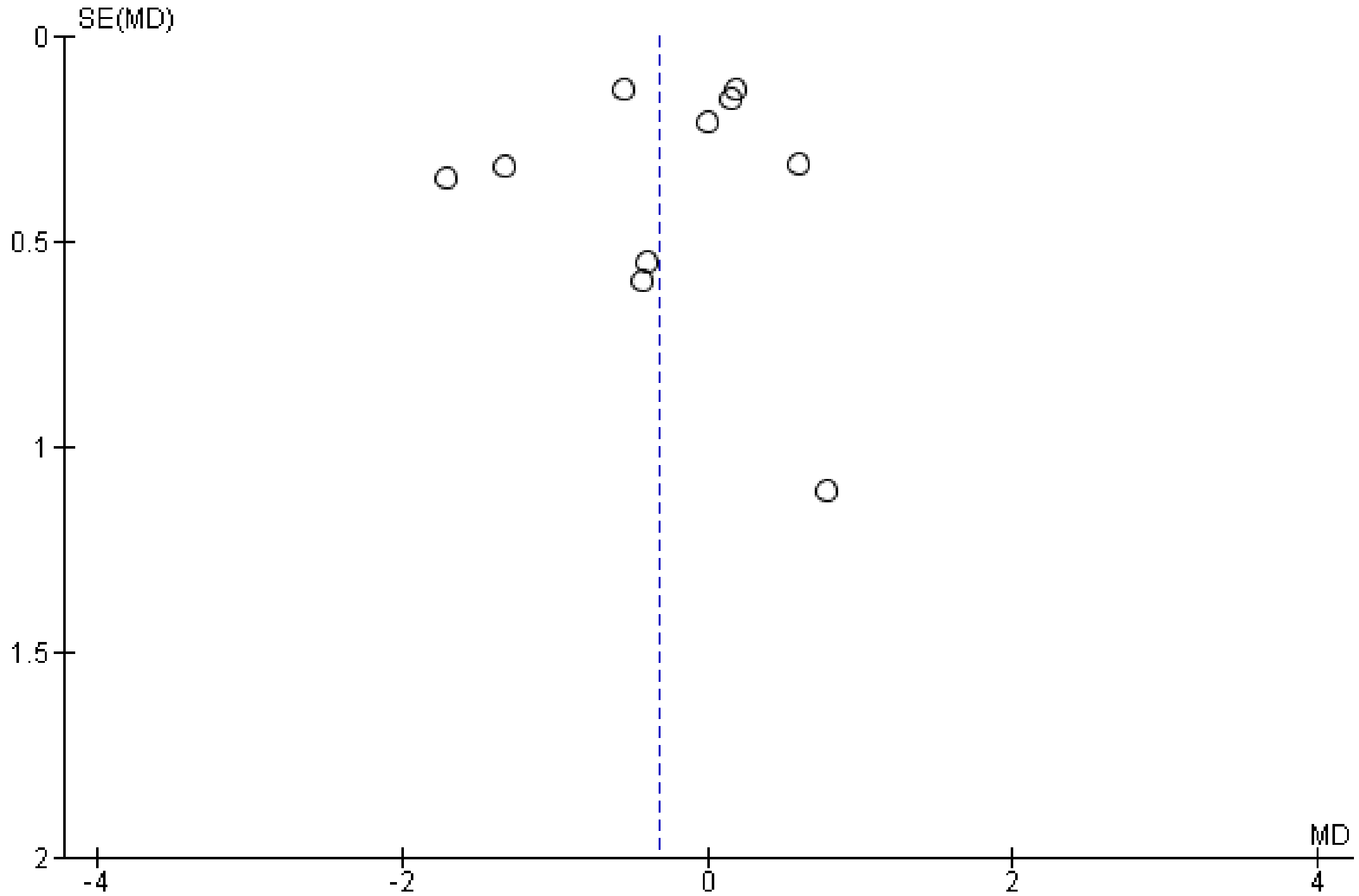

Figure e10: Funnel plot of comparison: HFNC vs CPAP, outcome: Length of PICU Stay (days)

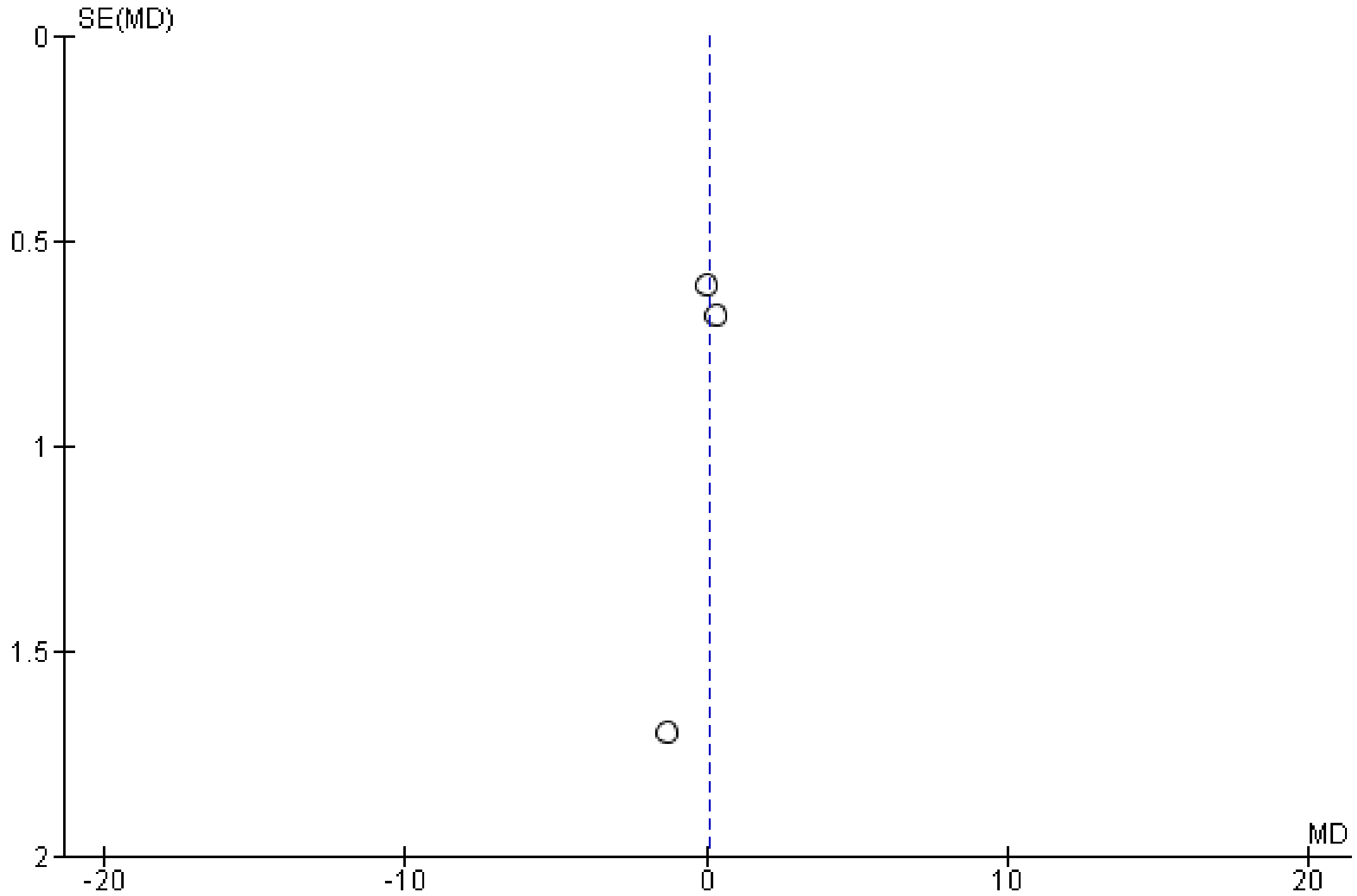

Figure e11: Funnel plot of comparison: HFNC vs LFNC, outcome: Length of PICU stay (days)

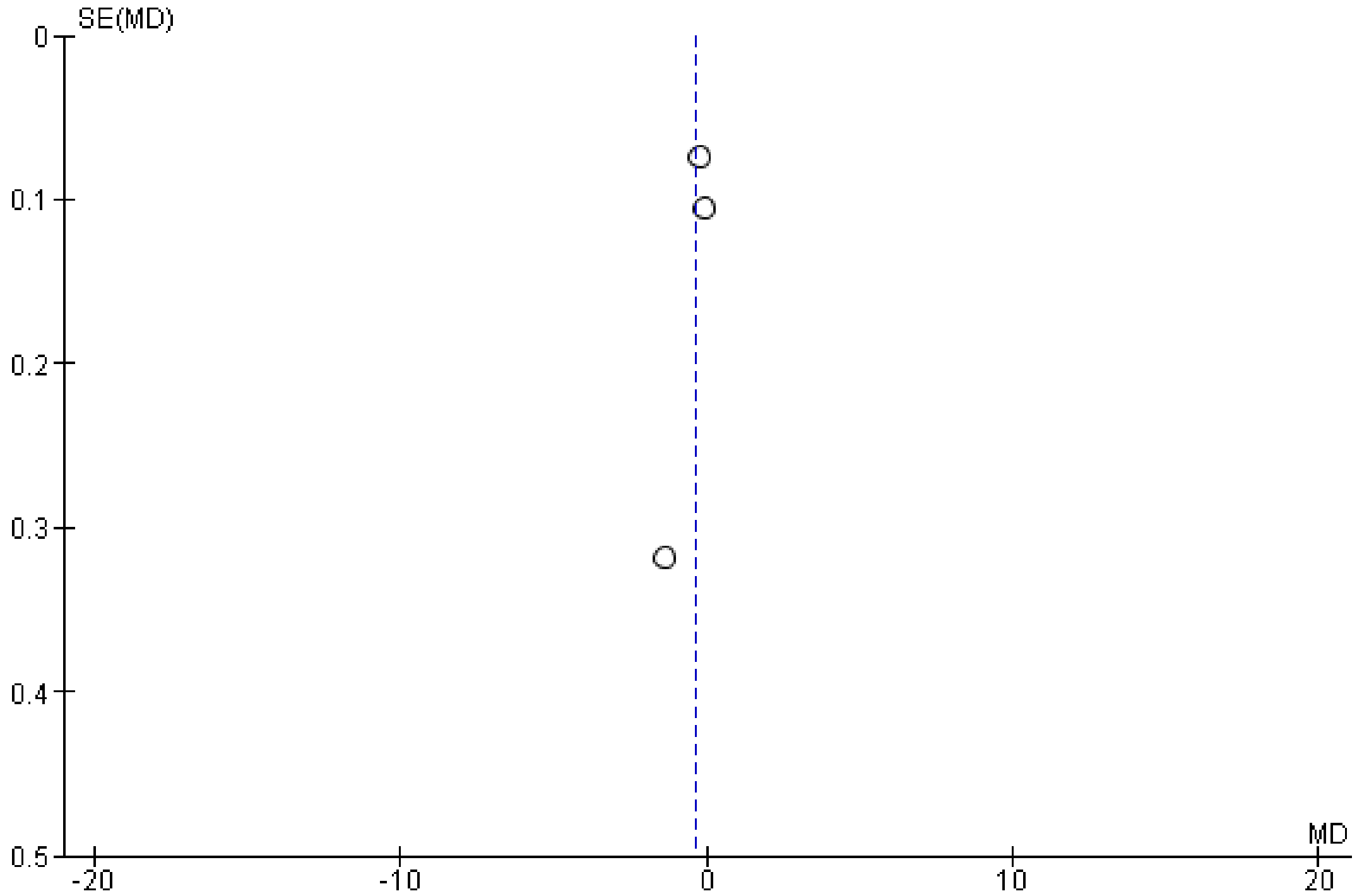

Figure e12: Funnel plot of comparison: HFNC vs LFNC, outcome: Total Oxygen Therapy (days)

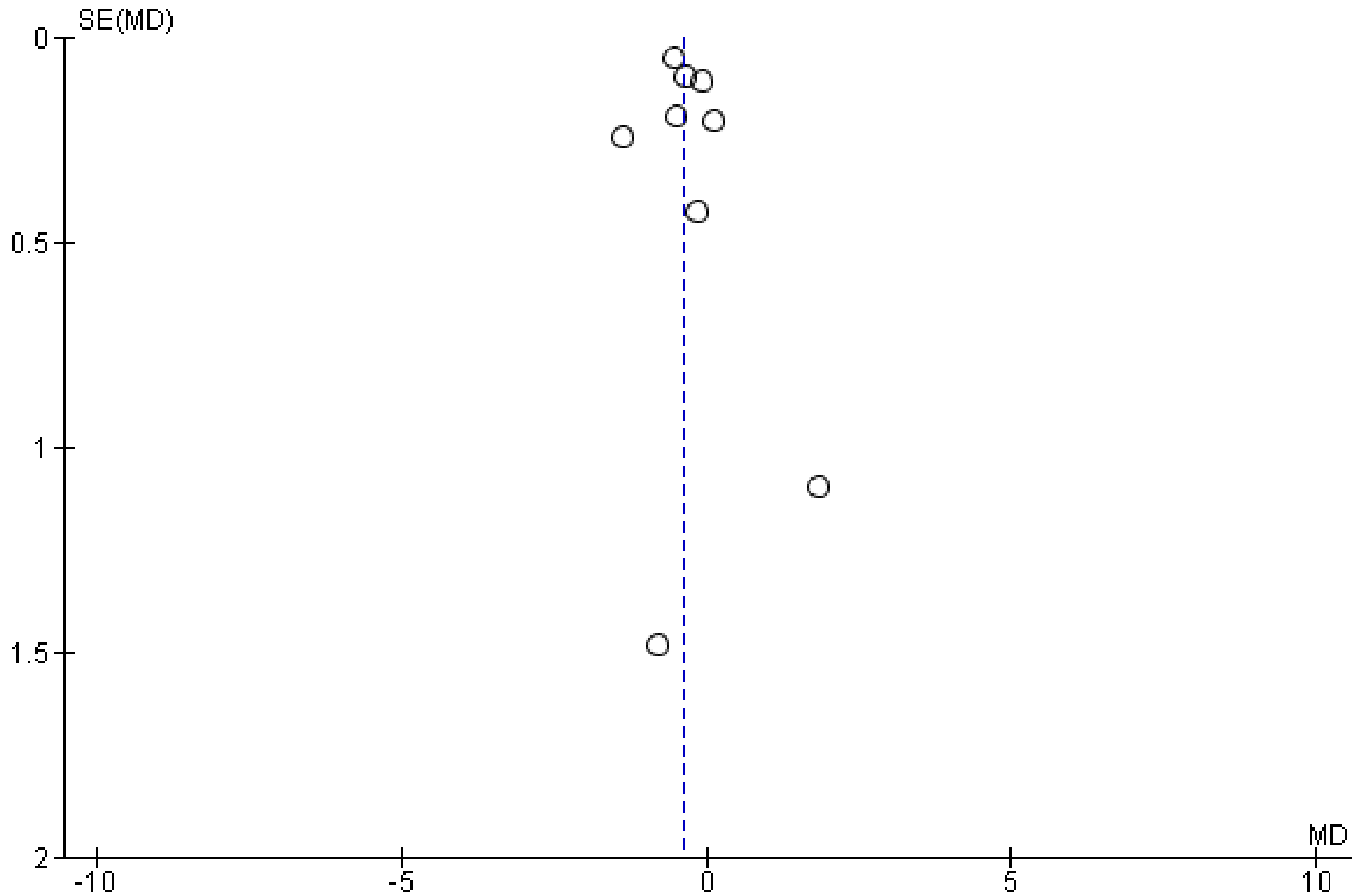

Figure e13: Forest plot of comparison: HFNC vs LFNC, outcome: Admission to PICU.

	HFNC		LFNC			Risk Ratio
**Study or Subgroup**	**Events**	**Total**	**Events**	**Total**	**Weight**	**M-H, Random, 95% CI**
Durand 2020	21	133	27	135	19.1%	0.79 [0.47, 1.32]
Eşki 2022	1	39	2	48	1.3%	0.62 [0.06, 6.54]
Franklin 2018	87	739	65	733	34.4%	1.33 [0.98, 1.80]
Franklin 2023	54	383	31	391	25.0%	1.78 [1.17, 2.70]
Kepreotes 2017	14	101	12	101	11.7%	1.17 [0.57, 2.40]
Kooiman 2023	8	55	6	52	6.8%	1.26 [0.47, 3.39]
Selvaraj 2022	0	40	2	40	0.8%	0.20 [0.01, 4.04]
Türe 2020	0	37	2	38	0.8%	0.21 [0.01, 4.14]
**Total (95% CI)**		**1527**		**1538**	**100.0%**	**1.22 [0.93, 1.60]**
Total events	185		147			

Heterogeneity: Tau² = 0.03; Chi² = 9.09, df = 7 (P = 0.25); I² = 23%

Test for overall effect: Z = 1.41 (P = 0.16)

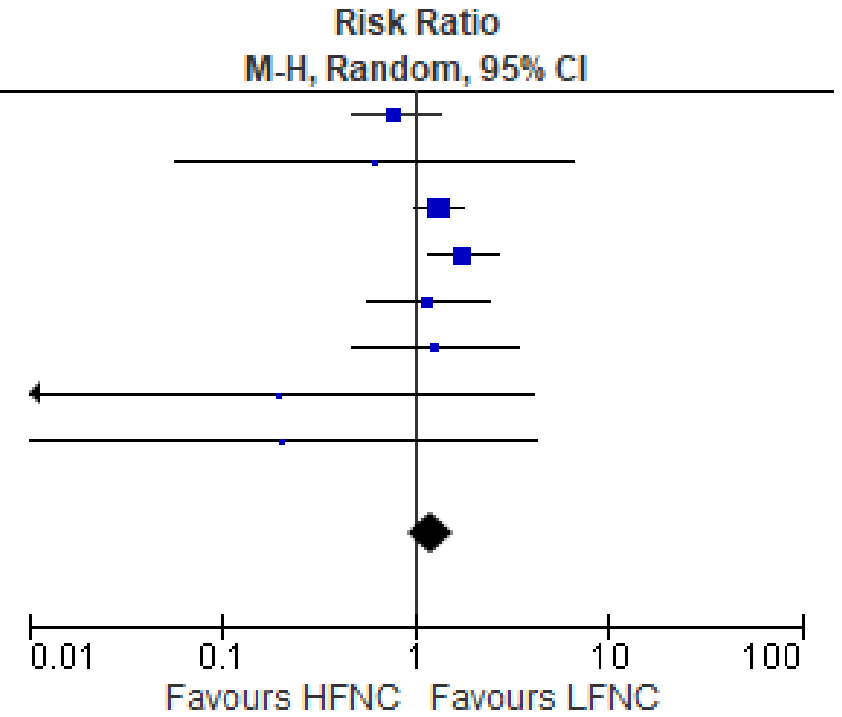

Figure e14: Forest plot of comparison: HFNC vs LFNC, outcome: Length of Hospital Stay (days).

	HFNC			LFNC				Mean Difference	Mean Difference
Study or Subgroup	Mean	SD	Total	Mean	SD	Total	Weight	IV, Random, 95% CI	IV, Random, 95% CI
Durand 2020	4.4	2.4	133	3.8	2.7	135	10.7%	0.60 [-0.01, 1.21]	
Ergul 2018	3.67	0.78	30	5	1.56	30	10.6%	-1.33 [-1.95, -0.71]	
Eşki 2022	5.33	2.31	39	5.73	2.83	48	7.1%	-0.40 [-1.48, 0.68]	
Franklin 2018	3.12	2.43	739	2.94	2.73	733	13.4%	0.18 [-0.08, 0.44]	
Franklin 2023	2.3	1.8	383	2.15	2.53	391	13.1%	0.15 [-0.16, 0.46]	
Hilliard 2011	7.5	2.67	11	6.72	2.16	8	2.8%	0.78 [-1.39, 2.95]	
Kepreotes 2017	2	1.5	101	2	1.5	101	12.4%	0.00 [-0.41, 0.41]	
Kooiman 2023	4.77	2.1	55	5.2	3.81	52	6.5%	-0.43 [-1.60, 0.74]	
Selvaraj 2022	3.65	1.46	40	5.35	1.657	40	10.1%	-1.70 [-2.38, -1.02]	
Türe 2020	1.29	0.5	37	1.84	0.66	38	13.4%	-0.55 [-0.81, -0.29]	
**Total (95% CI)**			**1568**			**1576**	**100.0%**	**-0.31 [-0.72, 0.09]**	

Heterogeneity: Tau² = 0.30; Chi² = 59.09, df = 9 (P < 0.00001); I² = 85%
Test for overall effect: Z = 1.52 (P = 0.13)

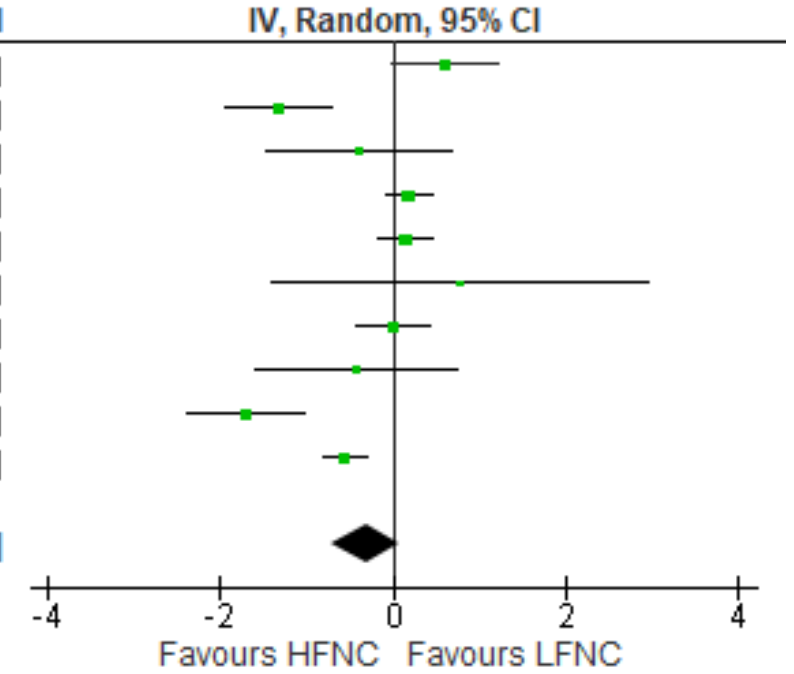

Figure e15: Forest plot of comparison: HFNC vs CPAP, outcome: Length of Hospital Stay (days).

	HFNC			CPAP				Mean Difference
Study or Subgroup	Mean	SD	Total	Mean	SD	Total	Weight	IV, Random, 95% CI
Borgi 2021	5.9	4.1	130	6.7	5.6	125	55.8%	-0.80 [-2.01, 0.41]
Cesar 2020	9.33	3.86	35	8.67	3.12	28	38.4%	0.66 [-1.06, 2.38]
Ramnaryan 2022	5.5	7.2	135	8.8	31.8	135	5.8%	-3.30 [-8.80, 2.20]
**Total (95% CI)**			**300**			**288**	**100.0%**	**-0.38 [-1.75, 0.98]**

Heterogeneity: Tau² = 0.49; Chi² = 2.94, df = 2 (P = 0.23); I² = 32%
Test for overall effect: Z = 0.55 (P = 0.58)

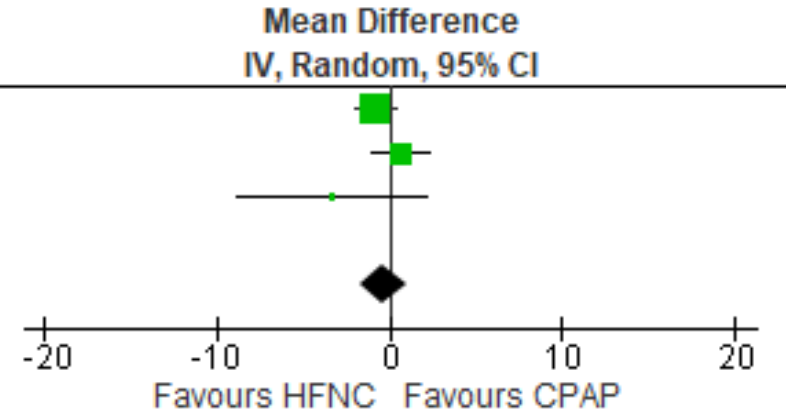

Figure e16: Forest plot of comparison: HFNC vs LFNC, outcome: Length of PICU stay (days).

	HFNC			LFNC				Mean Difference
**Study or Subgroup**	**Mean**	**SD**	**Total**	**Mean**	**SD**	**Total**	**Weight**	**IV, Random, 95% CI**
Ergul 2018	2.67	0.78	30	4	1.56	30	20.1%	-1.33 [-1.95, -0.71]
Franklin 2018	2.63	1.7	739	2.72	2.31	733	38.7%	-0.09 [-0.30, 0.12]
Franklin 2023	1.55	1.13	383	1.78	0.91	391	41.1%	-0.23 [-0.37, -0.09]
**Total (95% CI)**			**1152**			**1154**	**100.0%**	**-0.40 [-0.78, -0.02]**

Heterogeneity: Tau² = 0.09; Chi² = 13.67, df = 2 (P = 0.001); I² = 85%
Test for overall effect: Z = 2.04 (P = 0.04)

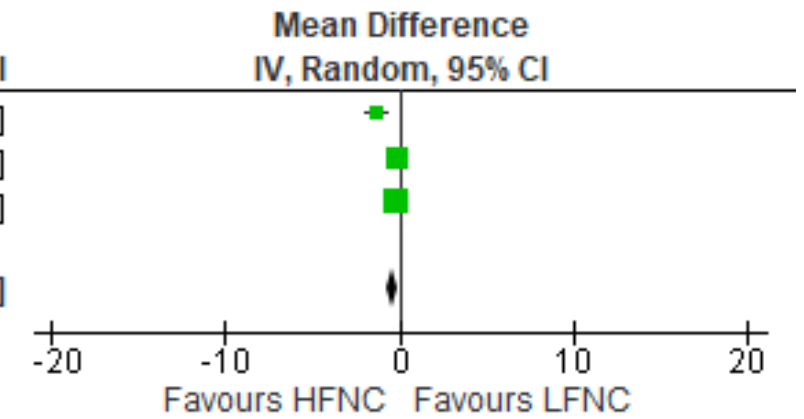

Figure e17: Forest plot of comparison: HFNC vs CPAP, outcome: Length of PICU Stay (days).

	HFNC			CPAP				Mean Difference
**Study or Subgroup**	**Mean**	**SD**	**Total**	**Mean**	**SD**	**Total**	**Weight**	**IV, Random, 95% CI**
Cesar 2020	5.67	3.09	35	5.33	2.34	28	24.9%	0.34 [-1.00, 1.68]
Milesi 2017	6.2	6	71	7.5	13	71	4.0%	-1.30 [-4.63, 2.03]
Ramnaryan 2022	3	2.6	141	3.7	5.8	136	39.5%	-0.70 [-1.77, 0.37]
Sarkar 2018	5	1.6	15	5	1.788	16	31.5%	0.00 [-1.19, 1.19]
**Total (95% CI)**			**262**			**251**	**100.0%**	**-0.24 [-0.91, 0.43]**

Heterogeneity: Tau² = 0.00; Chi² = 1.98, df = 3 (P = 0.58); I² = 0%
Test for overall effect: Z = 0.72 (P = 0.47)

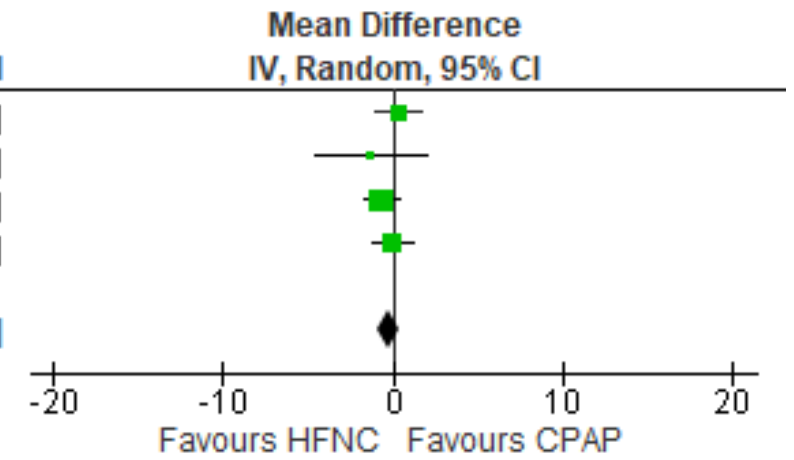

Figure e18: Forest plot of comparison: HFNC vs LFNC, outcome: Total Oxygen Therapy (days).

	HFNC			LFNC				Mean Difference
**Study or Subgroup**	**Mean**	**SD**	**Total**	**Mean**	**SD**	**Total**	**Weight**	**IV, Random, 95% CI**
Durand 2020	1.7	17	133	2.5	2	135	0.7%	-0.80 [-3.71, 2.11]
Ergul 2018	2.36	0.97	30	3.73	0.94	30	11.5%	-1.37 [-1.85, -0.89]
Eşki 2022	0.74	0.83	39	1.23	1.01	48	13.5%	-0.49 [-0.88, -0.10]
Franklin 2018	1.81	2.18	739	1.87	2.09	733	17.1%	-0.06 [-0.28, 0.16]
Hilliard 2011	5.4	2.04	11	3.58	2.56	8	1.3%	1.82 [-0.32, 3.96]
Kepreotes 2017	1.06	1.79	101	0.96	1.06	101	13.1%	0.10 [-0.31, 0.51]
Kooiman 2023	3.5	1.97	55	3.67	2.44	52	6.3%	-0.17 [-1.01, 0.67]
Selvaraj 2022	0.58	0.26	40	1.11	0.2	40	18.9%	-0.53 [-0.63, -0.43]
Türe 2020	0.71	0.4	37	1.05	0.44	38	17.6%	-0.34 [-0.53, -0.15]
**Total (95% CI)**			**1185**			**1185**	**100.0%**	**-0.37 [-0.63, -0.12]**

Heterogeneity: Tau² = 0.09; Chi² = 41.46, df = 8 (P < 0.00001); I² = 81%
Test for overall effect: Z = 2.85 (P = 0.004)

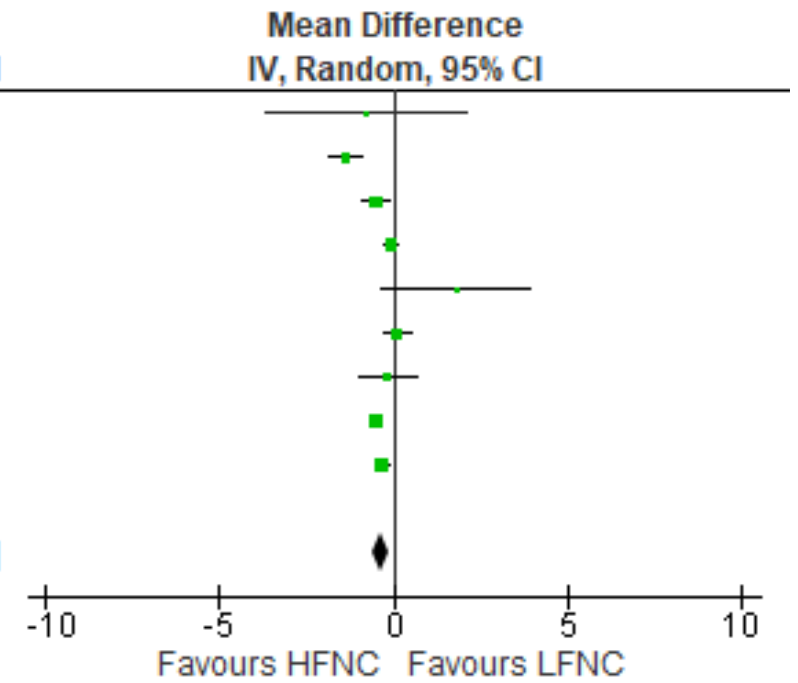

Figure e19. **Forest plot of comparison: HFNC vs CPAP, outcome: Need for mechanical ventilation – sensitivity analysis**

	HFNC		CPAP			Risk Ratio
**Study or Subgroup**	**Events**	**Total**	**Events**	**Total**	**Weight**	**M-H, Random, 95% CI**
Borgi 2021	29	130	37	125	65.2%	0.75 [0.50, 1.15]
Milesi 2017	5	71	3	71	5.9%	1.67 [0.41, 6.71]
Ramnaryan 2022	17	142	17	138	28.9%	0.97 [0.52, 1.82]
**Total (95% CI)**		**343**		**334**	**100.0%**	**0.85 [0.61, 1.19]**
Total events	51		57			

Heterogeneity: Tau² = 0.00; Chi² = 1.40, df = 2 (P = 0.50); I² = 0%

Test for overall effect: Z = 0.94 (P = 0.35)

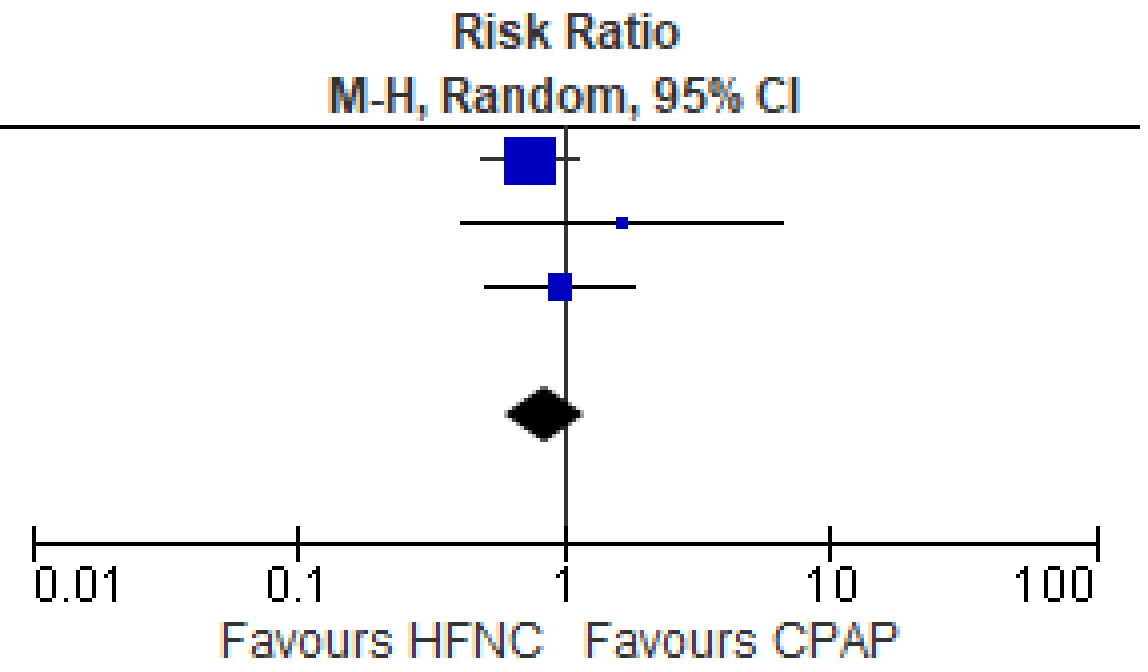

Figure e20. **Funnel plot of comparison: HFNC vs CPAP, outcome: Need for mechanical ventilation – sensitivity analysis**

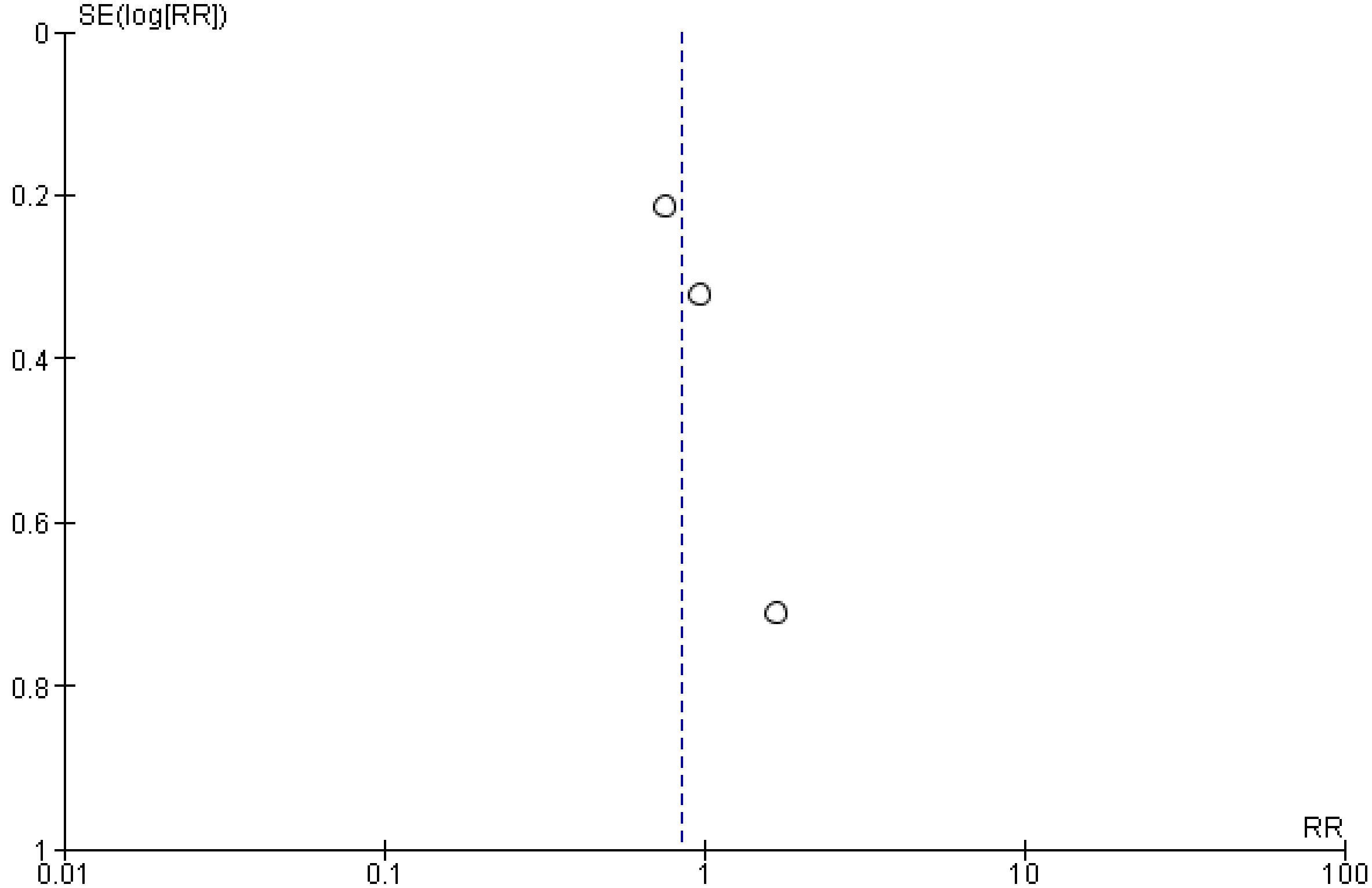

Figure e21**. Forest plot of comparison: HFNC vs CPAP, outcome: Treatment failure – sensitivity analysis**

	HFNC		CPAP			Risk Ratio
**Study or Subgroup**	**Events**	**Total**	**Events**	**Total**	**Weight**	**M-H, Random, 95% CI**
Borgi 2021	64	130	37	125	32.2%	1.66 [1.21, 2.29]
Milesi 2017	36	71	22	71	28.8%	1.64 [1.08, 2.48]
Ramnaryan 2022	48	144	56	142	32.7%	0.85 [0.62, 1.15]
Vahlkvist 2019	2	22	4	28	6.3%	0.64 [0.13, 3.16]
**Total (95% CI)**		**367**		**366**	**100.0%**	**1.25 [0.81, 1.94]**
Total events	150		119			

Heterogeneity: Tau² = 0.13; Chi² = 11.51, df = 3 (P = 0.009); I² = 74%
Test for overall effect: Z = 1.00 (P = 0.32)

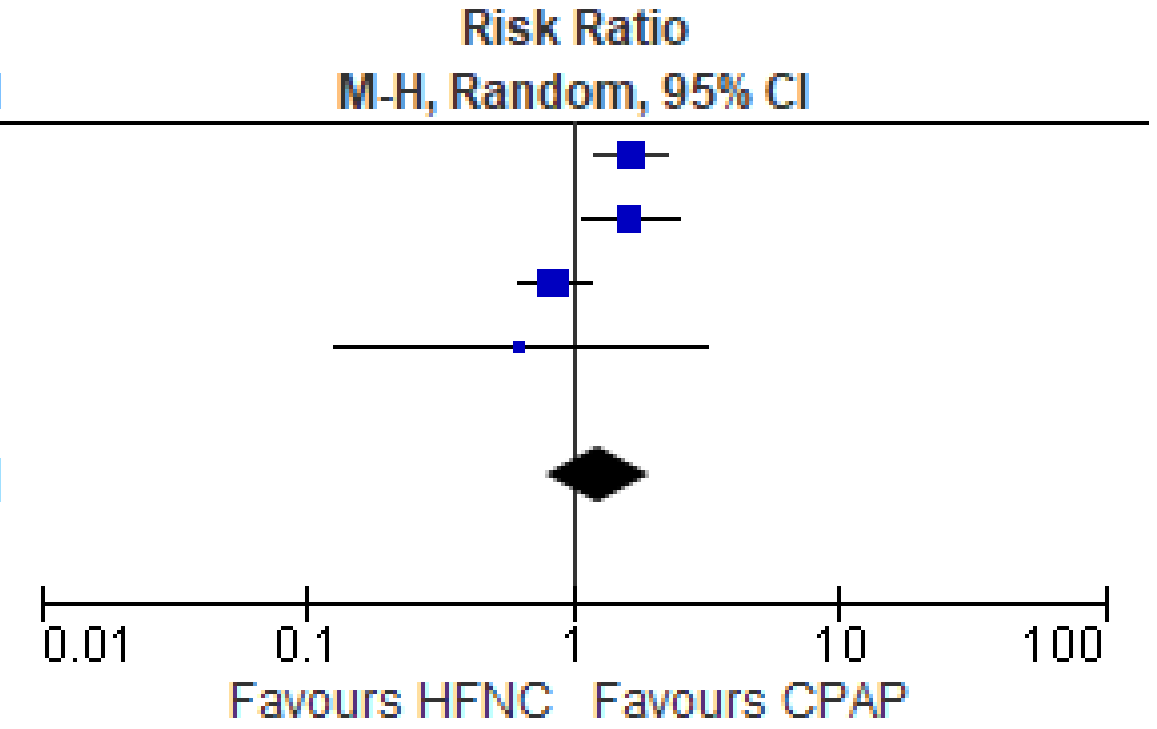

Figure e22. **Funnel plot of comparison: HFNC vs CPAP, outcome: Treatment failure – sensitivity analysis**

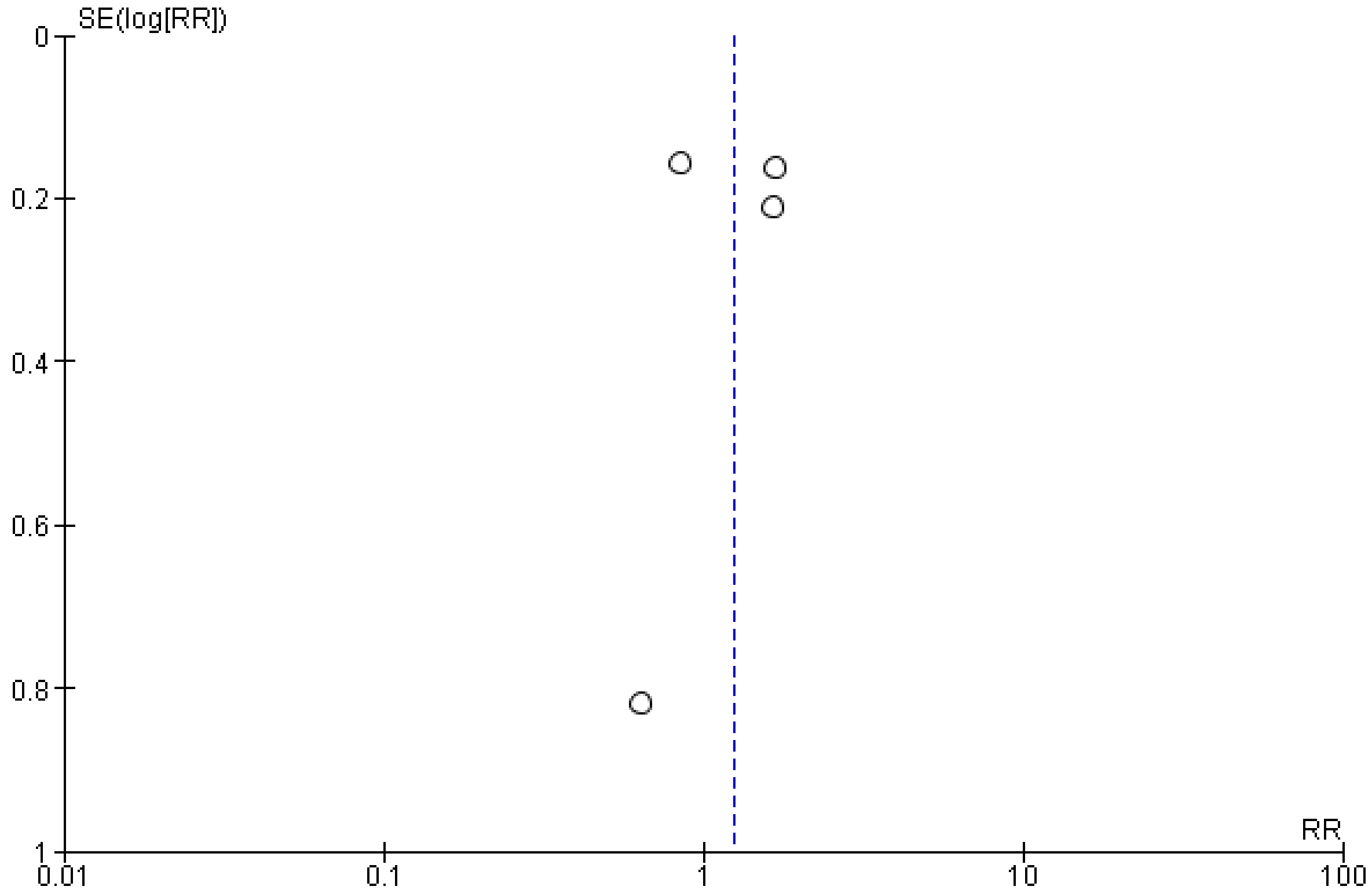

Figure e23. **Forest plot of comparison: HFNC vs LFNC, outcome: Need for mechanical ventilation – sensitivity analysis**

	Favours HFNC		LFNC			Risk Ratio	Risk Ratio
**Study or Subgroup**	**Events**	**Total**	**Events**	**Total**	**Weight**	**M-H, Random, 95% CI**	**M-H, Random, 95% CI**
Durand 2020	0	133	0	135		Not estimable	
Ergul 2018	0	30	0	30		Not estimable	
Eşki 2022	1	39	2	48	11.0%	0.62 [0.06, 6.54]	
Franklin 2018	8	739	4	733	25.0%	1.98 [0.60, 6.56]	
Franklin 2023	0	383	0	391		Not estimable	
Guna 2023	4	50	15	50	28.2%	0.27 [0.10, 0.75]	
Kooiman 2023	8	55	6	53	29.0%	1.28 [0.48, 3.45]	
Selvaraj 2022	0	40	1	40	6.8%	0.33 [0.01, 7.95]	
Türe 2020	0	37	0	38		Not estimable	
**Total (95% CI)**		**1506**		**1518**	**100.0%**	**0.77 [0.31, 1.90]**	
Total events	21		28				

Heterogeneity: Tau² = 0.47; Chi² = 7.82, df = 4 (P = 0.10); I² = 49%

Test for overall effect: Z = 0.56 (P = 0.58)

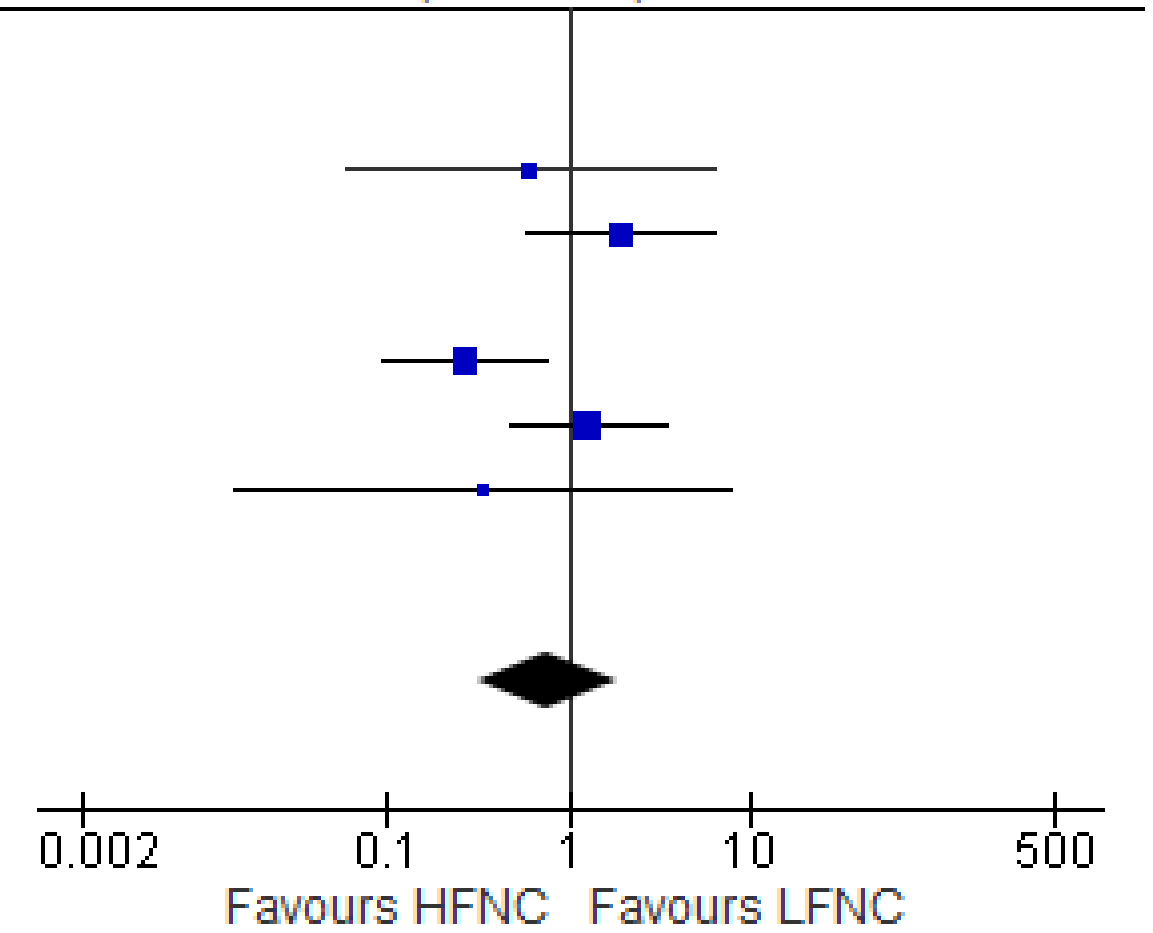

Figure e24. **Funnel plot of comparison: HFNC vs LFNC, outcome: Need for mechanical ventilation – sensitivity analysis**

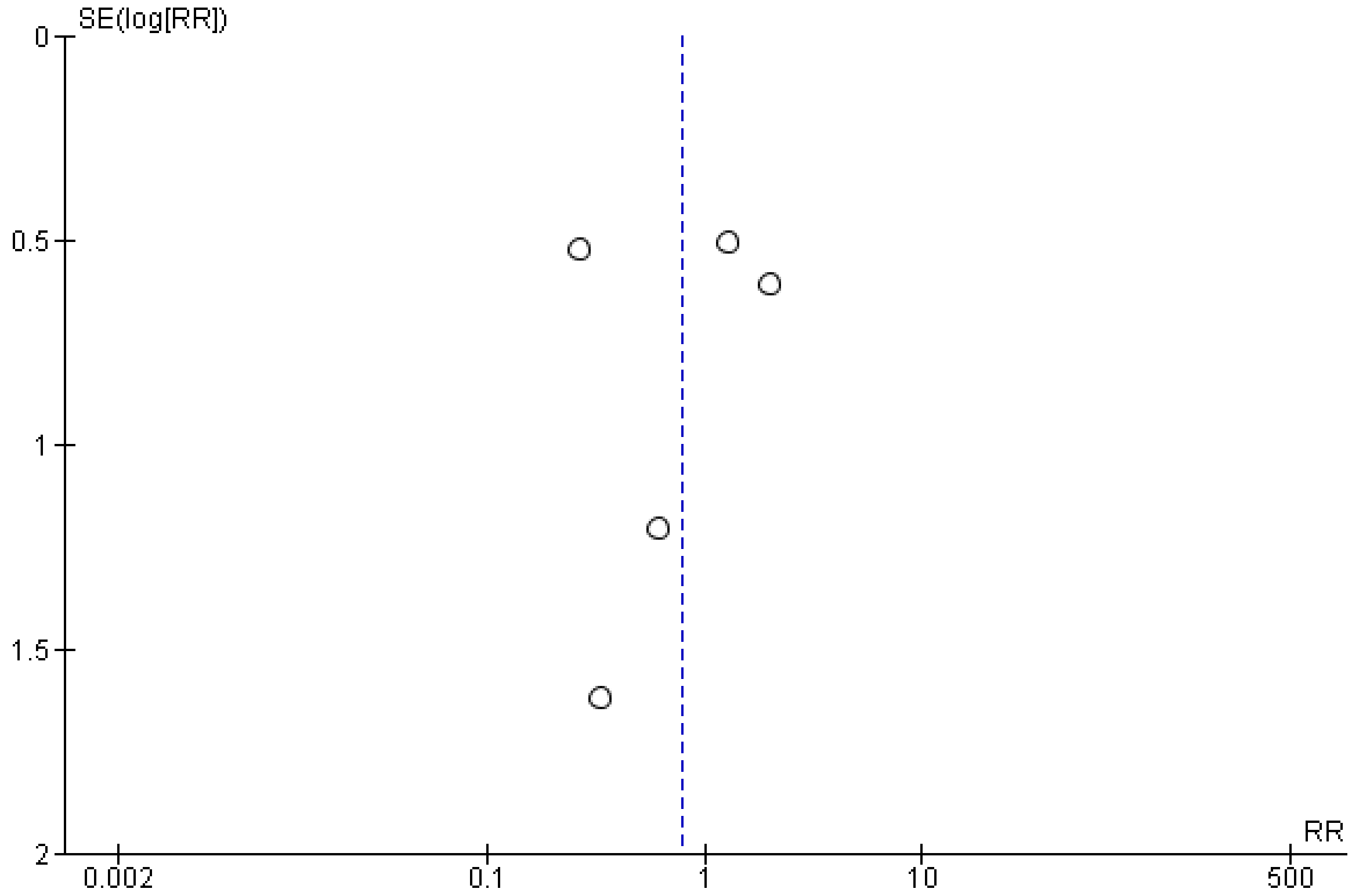

Figure e25. **Forest plot of comparison: HFNC vs LFNC, outcome: Treatment failure – sensitivity analysis**

	HFNC		LFNC			Risk Ratio
**Study or Subgroup**	**Events**	**Total**	**Events**	**Total**	**Weight**	**M-H, Random, 95% CI**
Durand 2020	19	133	27	135	15.5%	0.71 [0.42, 1.22]
Ergul 2018	0	30	7	30	4.7%	0.07 [0.00, 1.12]
Eşki 2022	1	39	10	48	7.3%	0.12 [0.02, 0.92]
Franklin 2018	87	739	167	733	16.7%	0.52 [0.41, 0.66]
Franklin 2023	196	383	103	391	16.8%	1.94 [1.60, 2.35]
Guna 2023	4	50	15	50	12.6%	0.27 [0.10, 0.75]
Kepreotes 2017	14	101	34	101	15.4%	0.41 [0.24, 0.72]
Selvaraj 2022	0	40	1	40	3.9%	0.33 [0.01, 7.95]
Türe 2020	1	37	7	38	7.1%	0.15 [0.02, 1.13]
**Total (95% CI)**		**1552**		**1566**	**100.0%**	**0.44 [0.22, 0.91]**
Total events	322		371			

Heterogeneity: Tau² = 0.79; Chi² = 107.42, df = 8 (P < 0.00001); I² = 93%
Test for overall effect: Z = 2.22 (P = 0.03)

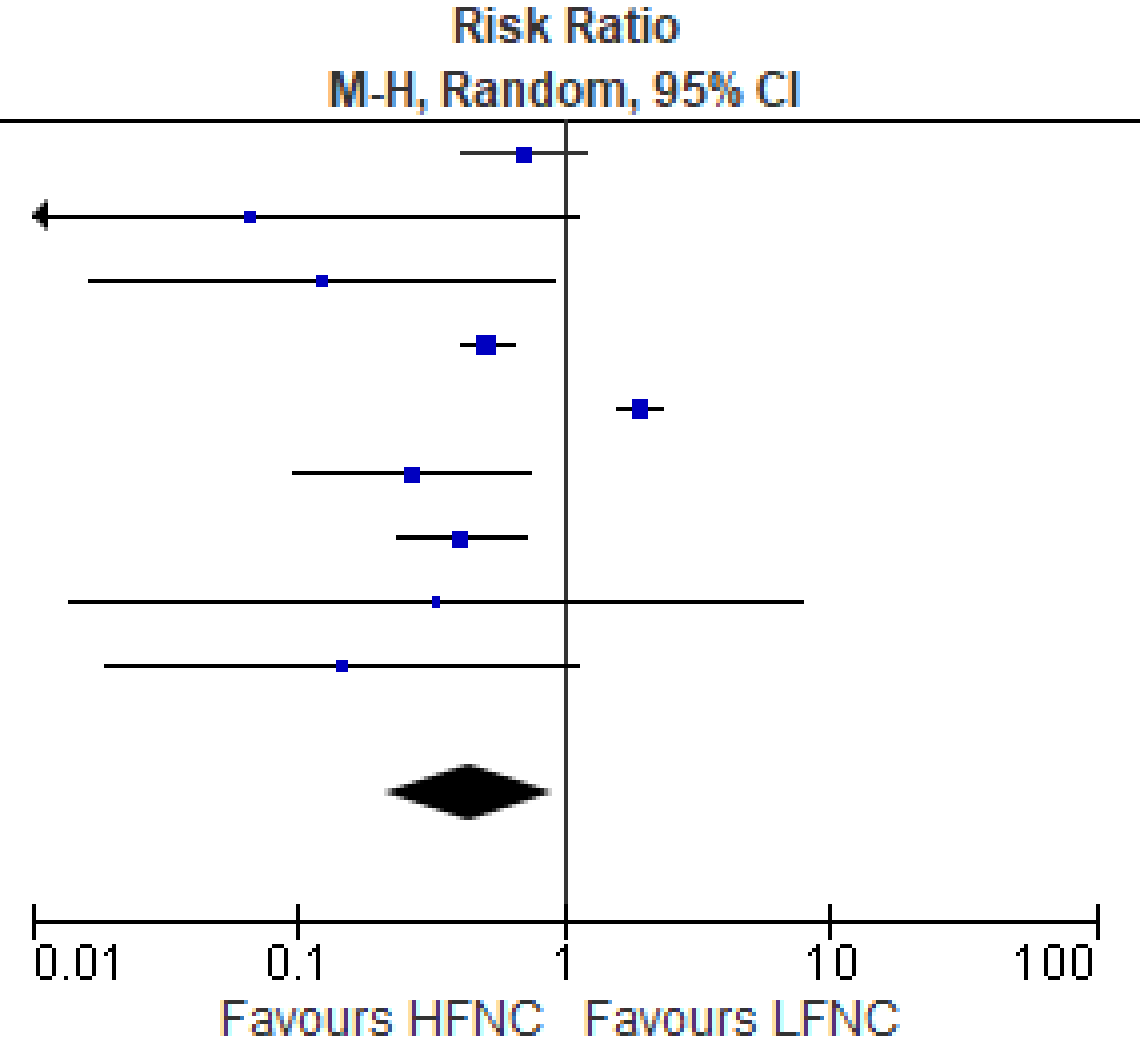

Figure e26. **Funnel plot of comparison: HFNC vs LFNC, outcome: Treatment failure – sensitivity analysis**

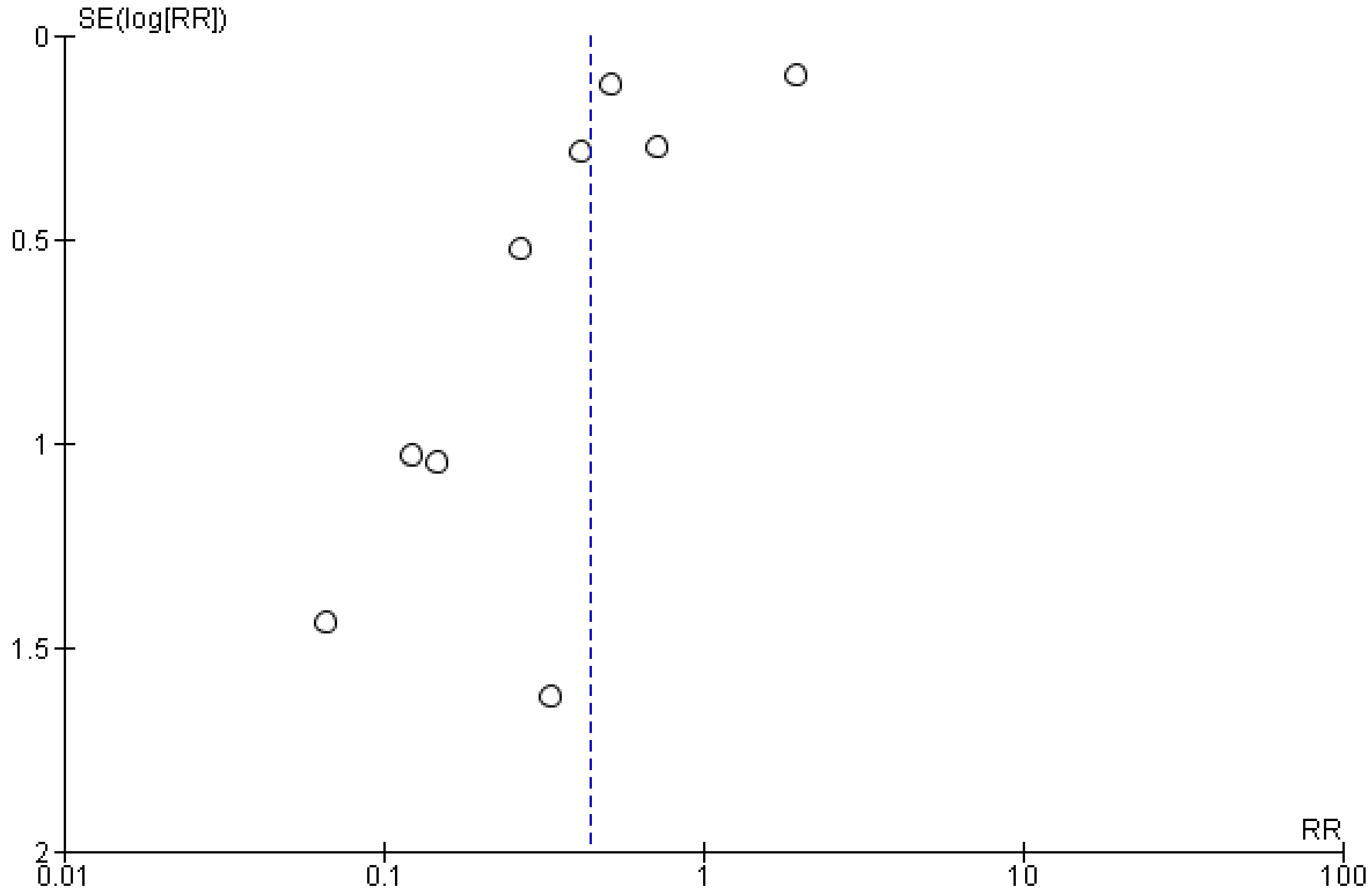

Figure e27. **Forest plot of comparison: HFNC vs CPAP, outcome: Length of Hospital Stay (days) – sensitivity analysis**

	HFNC			CPAP				Mean Difference
Study or Subgroup	Mean	SD	Total	Mean	SD	Total	Weight	IV, Random, 95% CI
Borgi 2021	5.9	4.1	130	6.7	5.6	125	95.4%	-0.80 [-2.01, 0.41]
Ramnaryan 2022	5.5	7.2	135	8.8	31.8	135	4.6%	-3.30 [-8.80, 2.20]
**Total (95% CI)**			**265**			**260**	**100.0%**	**-0.92 [-2.10, 0.27]**

Heterogeneity: Tau² = 0.00; Chi² = 0.76, df = 1 (P = 0.38); I² = 0%
Test for overall effect: Z = 1.52 (P = 0.13)

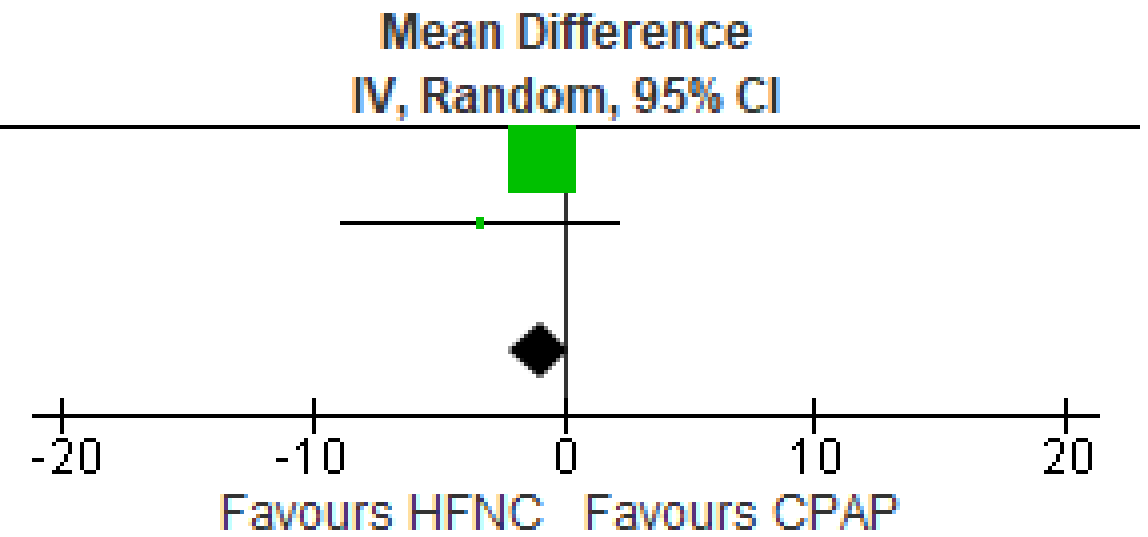

Figure e28. **Funnel plot of comparison: HFNC vs CPAP, outcome: Length of Hospital Stay (days) – sensitivity analysis**

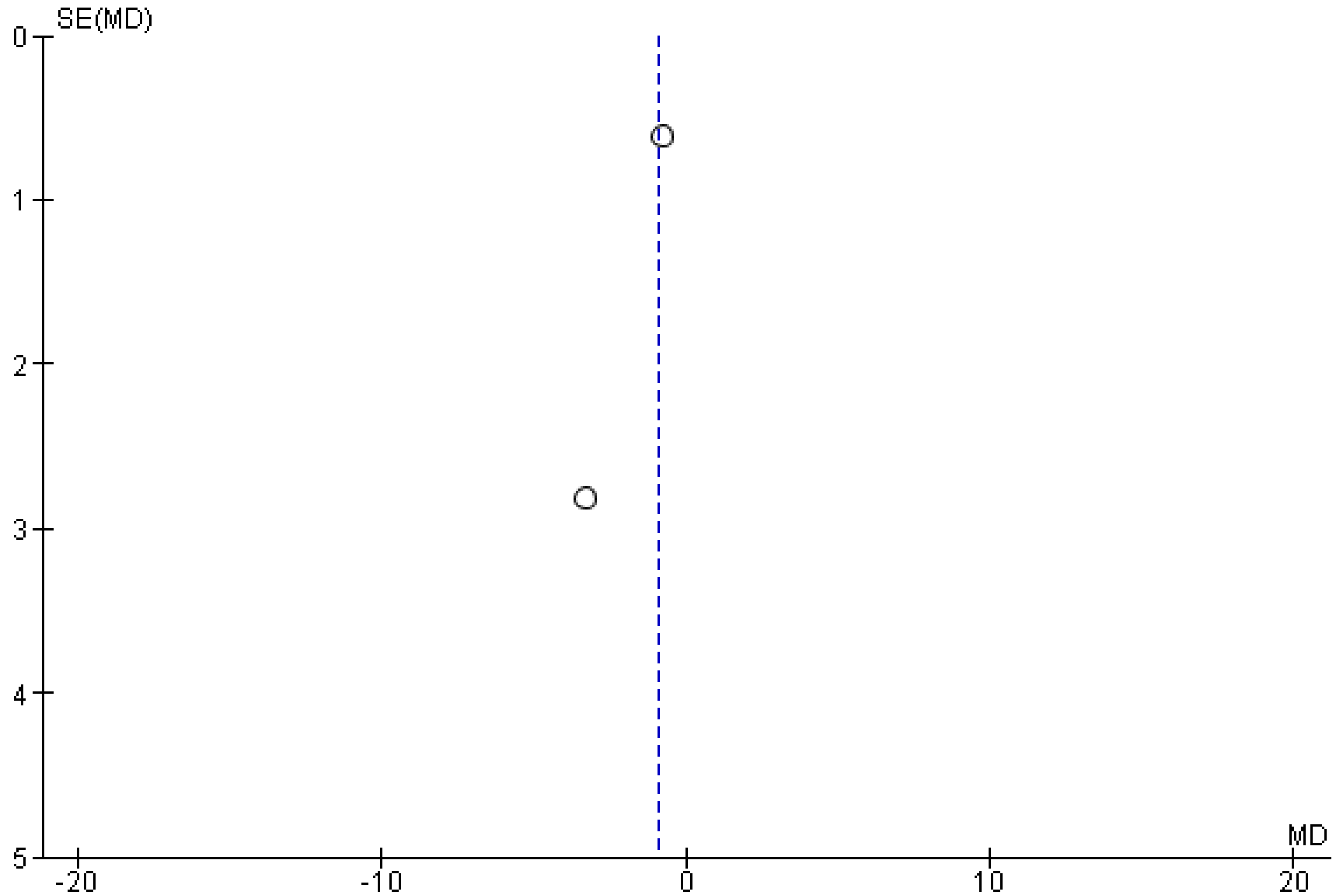

Figure e29. **Forest plot of comparison: HFNC vs CPAP, outcome: Length of PICU Stay (days) – sensitivity analysis**

	HFNC			CPAP				Mean Difference
**Study or Subgroup**	**Mean**	**SD**	**Total**	**Mean**	**SD**	**Total**	**Weight**	**IV, Random, 95% CI**
Milesi 2017	6.2	6	71	7.5	13	71	9.3%	-1.30 [-4.63, 2.03]
Ramnaryan 2022	3	2.6	141	3.7	5.8	136	90.7%	-0.70 [-1.77, 0.37]
**Total (95% CI)**			**212**			**207**	**100.0%**	**-0.76 [-1.77, 0.26]**

Heterogeneity: Tau² = 0.00; Chi² = 0.11, df = 1 (P = 0.74); I² = 0%

Test for overall effect: Z = 1.46 (P = 0.14)

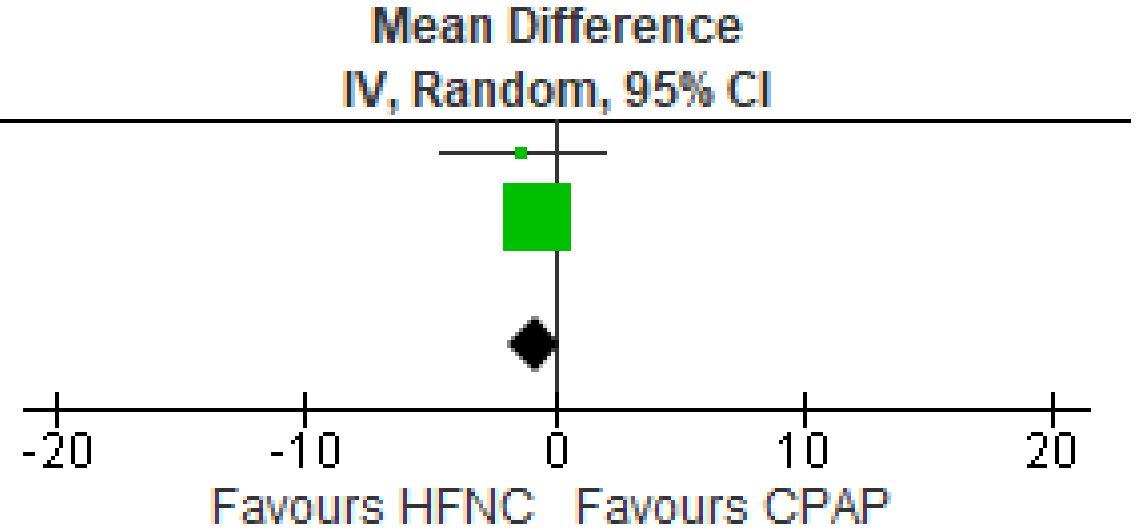

Figure e30. **Funnel plot of comparison: HFNC vs CPAP, outcome: Length of PICU Stay (days) – sensitivity analysis**

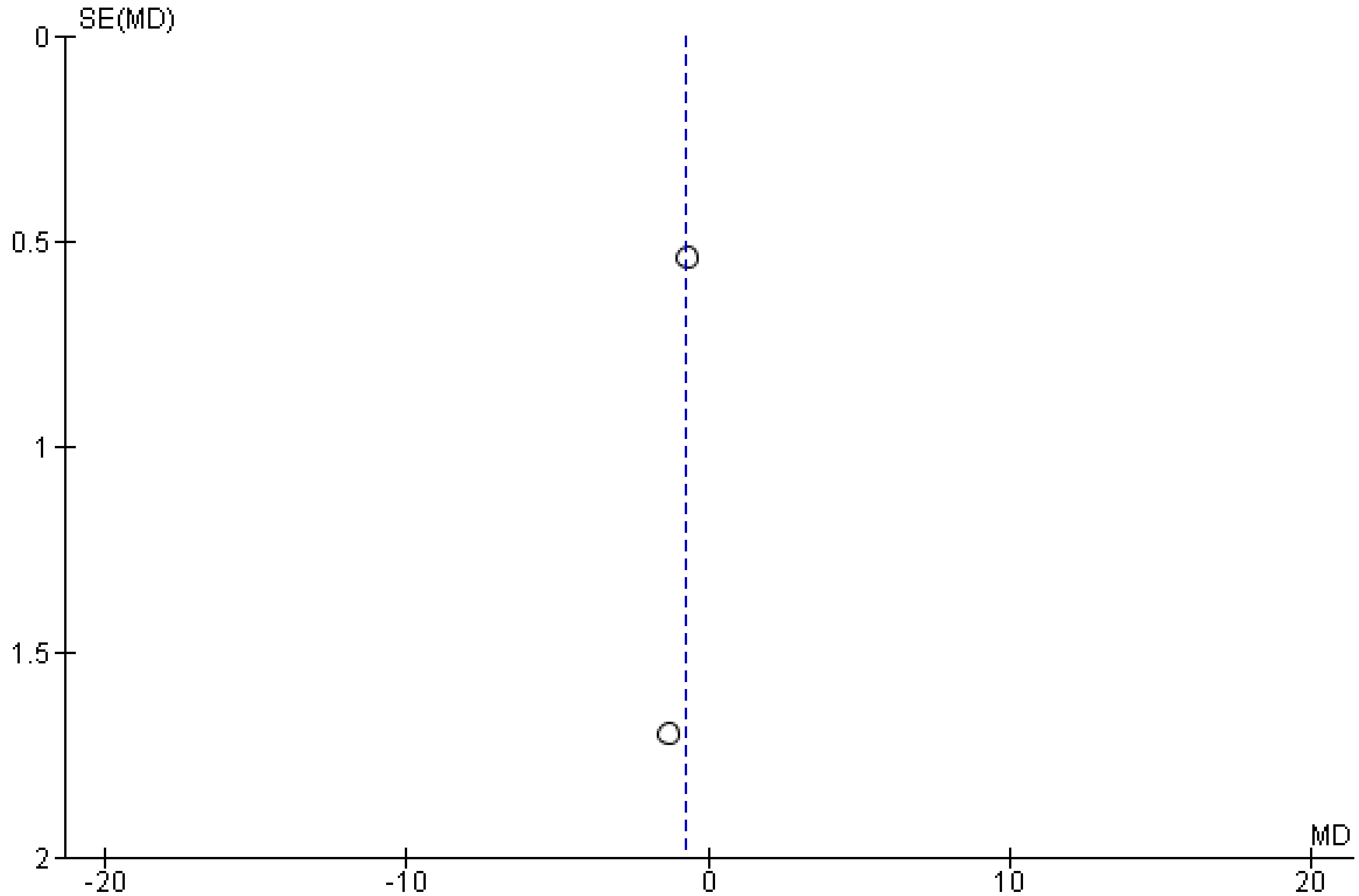

Supplement: Supplementary file 1 [file ERR-0223-2025.SUPPLEMENT.pdf]
